# Comparison of Serum, Plasma, and Liver Zinc Measurements by AAS, ICP-OES, and ICP-MS in Diverse Laboratory Settings

**DOI:** 10.1007/s12011-021-02883-z

**Published:** 2021-08-28

**Authors:** Andrew G. Hall, Janet C. King, Christine M. McDonald

**Affiliations:** 1 Benioff Children’s Hospitals; Children’s Hospital Oakland Research Institute, University of California San Francisco, Oakland, CA USA; 2 Department of Nutritional Sciences and Toxicology, University of California Berkeley, Berkeley, CA USA; 3 Department of Pediatrics, School of Medicine, University of California San Francisco, San Francisco, CA USA

**Keywords:** Plasma zinc, Zinc deficiency, Accuracy, Precision, Harmonization

## Abstract

Progress improving zinc nutrition globally is slowed by limited understanding of population zinc status. This challenge is compounded when small differences in measurement can bias the determination of zinc deficiency rates. Our objective was to evaluate zinc analytical accuracy and precision among different instrument types and sample matrices using a standardized method. Participating laboratories analyzed zinc content of plasma, serum, liver samples, and controls, using a standardized method based on current practice. Instrument calibration and drift were evaluated using a zinc standard. Accuracy was evaluated by percent error vs. reference, and precision by coefficient of variation (CV). Seven laboratories in 4 countries running 9 instruments completed the exercise: 4 atomic absorbance spectrometers (AAS), 1 inductively coupled plasma optical emission spectrometer (ICP-OES), and 4 ICP mass spectrometers (ICP-MS). Calibration differed between individual instruments up to 18.9% (*p* < 0.001). Geometric mean (95% CI) percent error was 3.5% (2.3%, 5.2%) and CV was 2.1% (1.7%, 2.5%) overall. There were no significant differences in percent error or CV among instrument types (*p* = 0.91, p = 0.15, respectively). Among sample matrices, serum and plasma zinc measures had the highest CV: 4.8% (3.0%, 7.7%) and 3.9% (2.9%, 5.4%), respectively (*p* < 0.05). When using standardized materials and methods, similar zinc concentration values, accuracy, and precision were achieved using AAS, ICP-OES, or ICP-MS. However, method development is needed for improvement in serum and plasma zinc measurement precision. Differences in calibration among instruments demonstrate a need for harmonization among laboratories.

**Supplementary Information:**

The online version contains supplementary material available at 10.1007/s12011-021-02883-z.

## Introduction

Nearly 20% of the global population is at risk of inadequate zinc intake [1]. Zinc is an essential micronutrient with unique roles in protein structure and as a cofactor in substrate binding and enzymatic activity. Due to these molecular roles, zinc contributes to broad range of functions including DNA transcription and repair, cell signaling, energy metabolism, immune and central nervous system function, and growth [2–4]. In the absence of a severe zinc deficiency, circulating zinc is highly conserved [5], and there is no sensitive and specific indicator of zinc status in individuals [2, 6]. The distribution of plasma or serum zinc concentration within a population does, however, allow the determination of population zinc status [6]. Therefore, plasma or serum zinc concentration is one of three recommended biomarkers for assessment of zinc status at the population level along with dietary zinc intake and the prevalence of stunting among children under 5 [2].

Future progress towards reducing zinc deficiency globally will depend on monitoring changes in plasma or serum zinc status. Apart from dietary zinc intake, a number of factors affect plasma or serum zinc concentration, including systemic inflammation, time of specimen collection, fasting status, and variations in the processing and handling of samples [2, 6–8]. To minimize the impact of some of these issues, recommendations have been made for the design of human studies assessing zinc status, and procedures for collecting samples for zinc analysis [6, 9].

The extent that analytical method and instrument may further contribute to variation in reported plasma or serum zinc values has seen limited study. The instruments typically used for zinc analysis vary in sensitivity and complexity of operation. They include atomic absorption spectrometers (AAS, flame or graphite furnace), inductively coupled plasma optical (atomic) emission spectrometers (ICP-OES/ICP-AES), and ICP mass spectrometers (ICP-MS). Our primary aim was an evaluation of accuracy and precision by instrument type and by sample type in diverse laboratory settings where zinc analysis of biological specimens from large surveys or clinical studies are typically conducted.

## Materials and Methods

We developed a standardized method based on a review of recent human studies, and instructed participating laboratories to implement the method for the analysis of a standardized set of samples and controls.

### Literature Review and Methods Reference Document

To determine the typical sample matrices, preparation methods, and analytical methods for the measurement of zinc in human studies, a literature review was conducted. Human studies published over the previous 5-years were identified in PubMed (National Center for Biotechnology Information, Bethesda, MD) using the single search term “zinc” and filters for human studies dated between February 2013 and February 2018. Studies written in English with abstracts reporting the measurement of zinc in human derived samples or foods were downloaded for further review. Articles without zinc analytical data in the full text were excluded. A detailed description of the literature review is provided in the accompanying online material (Supplemental Appendix A).

Three laboratories prominent in the literature review were contacted and their zinc analysis protocol(s) requested: Laboratory of Human Nutrition at the Institute of Food, Nutrition and Health, Swiss Federal Institute of Technology (Zurich, Switzerland); Section of Pediatric Nutrition, University of Colorado School of Medicine (Aurora, CO); and Children’s Hospital Oakland Research Institute (CHORI), UCSF Benioff Children’s Hospitals (Oakland, CA). Methodological elements of zinc analysis protocols from these laboratories, in addition to protocols provided by the Centers for Disease Control and Prevention (CDC, Atlanta, GA) [10] and the United States Department of Agriculture (USDA, Washington, DC) [11], were reviewed and assembled into a Methods Reference Document (Supplemental Appendix B) of techniques for determining zinc concentration in samples from human studies using AAS, ICP-OES, and ICP-MS. The methods document was sent to the three laboratories for review and revised according to their feedback.

### ***Laboratory Exercise***

A laboratory exercise was designed for comparison of accuracy and precision of zinc concentration measurements among different instruments and sample matrices selected based on the literature review. The laboratory exercise was designed to minimize variability due to sample preparation, reagent quality, and zinc reference material. In addition to the Methods Reference Document, specific instructions for instrument calibration, sample preparation, and a standard set of materials for analysis were included.

Laboratories in low- and middle-income countries that had previously conducted zinc analysis studies for national-level surveys, and laboratories that had contributed protocols for inclusion in the Methods Reference Document, were invited to participate in the laboratory exercise. Participating laboratories were instructed to use the identical sets of materials and supplies assembled at CHORI and shipped to each laboratory. These included trace element analysis grade 68–70% nitric acid (Omnitrace, EMD Millipore, Burlington, MA; or BDH Aristar Plus, VWR International, Radnor, PA), ultrapure water (Omnitrace Ultra, EMD Millipore, Burlington, MA), and filter pipette tips (VWR International, Radnor, PA) to minimize particulate contamination from the pipette mechanism.

Laboratories calibrated their instruments using the provided Standard Reference Material (SRM) 3168a zinc in 10% nitric acid (National Institute of Standards and Technology (NIST), Gaithersburg, MD). To detect potential differences in calibration, and to assess instrument drift during the analysis, a dilute solution of SRM 3168a, containing 12.5 µg zinc/dL in 5% nitric acid, was prepared in bulk at CHORI using the same ultrapure water and nitric acid provided to the participating laboratories.

Sample matrices included human serum and plasma, and powdered bovine liver (representative of prepared food composites). Unknowns (i.e., samples without a known value) for each, as well as reference materials with certified values, were included for analysis. Unknowns or reference materials were dispensed into vials at CHORI prior to distribution to the laboratories as follows: All vials were acid-washed prior to dispensing. Human plasma and human serum unknowns (Zenbio, Research Triangle Park, NC) were shipped unfrozen on cold pack from North Carolina to CHORI overnight the day of collection, mixed and aliquoted into screw-cap polypropylene vials, and then frozen. The liver unknown, food grade powdered bovine liver (CurEase, McEwen, TN), was mixed for 5 min using a food processor (Cuisinart, Stanford, CT) prior to dispensing into polypropylene screwcap vials.

Reference materials included SRM 1950 human plasma and SRM 1577c powdered bovine liver (NIST, Gaithersburg, MD), Seronorm Trace Elements in Human Serum Levels 1 and 2 (SERO AS, Billingstad, Norway), and a custom UTAK human plasma containing no added zinc (UTAK Laboratories, Valencia, CA). The SRM 1950 serum was distributed to each laboratory frozen in the sealed 1-mL glass ampoules with rubber stoppers, as provided by NIST. Lyophilized Seronorm serum and UTAK plasma were reconstituted at CHORI using ultrapure water (Omnitrace Ultra, EMD Millipore, Burlington, MA) according to the manufacturer’s instructions, and dispensed into screw cap polypropylene vials. The SRM 1577c bovine liver was also dispensed into screwcap polypropylene vials prior to distribution to the laboratories.

All sera and plasma were shipped on dry ice from CHORI to each participating laboratory. Zinc solutions, powdered liver, ultrapure water, and pipette tips were shipped separately from CHORI at ambient temperature. The trace element grade nitric acid was shipped directly from the supplier to each participating laboratory. To minimize the potential for bias, individual laboratories were not informed of the known zinc values of any of the materials sent, with the exception of the pre-diluted zinc standard, reported as falling within a range between 10 and 15 µg zinc/dL. Laboratories measured the zinc concentration of the pre-diluted zinc solution in triplicate at the beginning and end of the analytical run, that of each reference material in triplicate, and each unknown 9 times. To minimize variability in viscosity that could affect instrument sampling flow rates, a simple protocol for digestion of serum and plasma in concentrated nitric acid was specified.

### ***Data Analysis***

Instrument calibration was based on the measurement of zinc concentration in the pre-diluted SRM 3168a zinc solution for each instrument. Percent drift was defined as the relative change in measurement of the zinc solution from start to end of the analytical run:

$$\% \mathrm{drift}= \frac{{\left[Zn\right]}_{\mathrm{end}}-{\left[Zn\right]}_{\mathrm{start}}}{{\left[Zn\right]}_{\mathrm{start}}}\times 100\%$$

Precision was evaluated for all reference materials and unknowns by determining the coefficient of variation (CV), i.e., sample mean divided by the sample standard deviation for each sample analyzed, expressed as a percentage:

$$\mathrm{CV}= \frac{s}{\overline{x} }\times 100\%$$

Accuracy was evaluated using % error and % bias. Percent error was defined as the absolute value of the relative difference between the zinc concentration measured, and the published (reference) zinc concentration for each reference material:

$$\% \mathrm{error}= \left|\frac{{\left[Zn\right]}_{\mathrm{measured}}-{\left[Zn\right]}_{\mathrm{reference}}}{{\left[Zn\right]}_{\mathrm{reference}}}\right|\times 100$$

Percent bias was defined as the relative difference between the zinc concentration measured, and the published (reference) zinc concentration for each reference material (maintaining the sign of the difference from reference):

$$\% \mathrm{bias}= \frac{{\left[Zn\right]}_{\mathrm{measured}}-{\left[Zn\right]}_{\mathrm{reference}}}{{\left[Zn\right]}_{\mathrm{reference}}}\times 100$$

The overall CV, % error, and % bias for a given instrument were determined as the mean (or geometric mean) of the respective indicator for all samples analyzed by each instrument. The overall CV, % error, and % bias for a given sample matrix was determined as the overall mean (or geometric mean) value for the respective sample matrix.

All data were tabulated; % error, % bias, and CV were calculated using Microsoft Excel 2010 (Microsoft, Redmond, WA). Descriptive statistics were calculated and statistical comparisons performed using SPSS 26 (IBM, Armonk, NY). Data were tested for normal distribution and descriptive statistics calculated prior to statistical comparison by unpaired *t*-test or ANOVA. Unless stated otherwise, all data are formatted in the text as “mean ± standard deviation” or, where not normally distributed, as “geometric mean (95% confidence interval).” ICP-OES was not included in comparisons between instrument types, since there was only one such instrument. Values for CV and % error were log-transformed to achieve normal distribution prior to statistical comparison. Statistically significant differences were defined as *p* < 0.05 for all comparisons. As the study was not designed to evaluate the accuracy and precision of individual laboratories, the individual laboratories and their individual instruments are not identified with respect to analytical results.

## Results

### ***Literature Review***

Detailed results of the literature review are provided in the supplemental material (Supplemental Appendix A). Briefly, out of 470 PubMed search hits, 134 peer-reviewed journal articles met the criteria for review. Zinc concentration was most frequently determined in serum (*n* = 69), plasma (*n* = 48), and food composites (*n* = 12). Instruments for zinc quantification included AAS (*n* = 78 AAS), ICP-MS (*n* = 22), and ICP-OES (*n* = 16). While 38 of the studies using AAS described the instrument as a flame AAS, only three described using a graphite furnace AAS. Seven studies used a plate reader or auto-analyzer for indirect zinc determination utilizing a zinc sensitive chemical dye or probe.

### ***Laboratory Exercise***

**Fig. 1:**
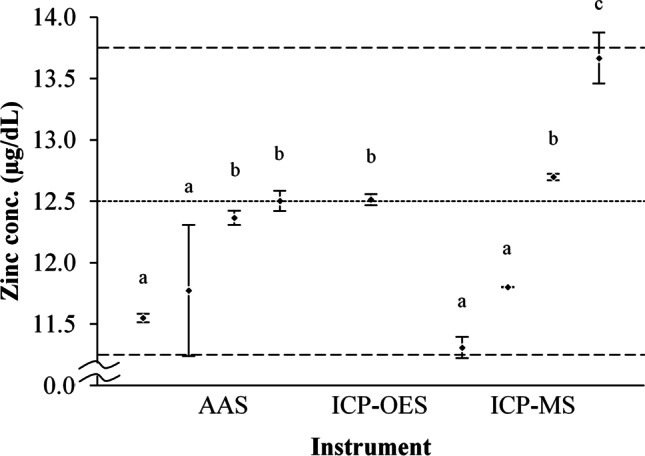
Instrument calibration. Pre-diluted SRM 3168a values from each instrument, with expected concentration (12.5 µg/dL, dotted horizontal line), and range of ± 10% from the expected value (horizontal dashed lines). Data are displayed as mean ± standard deviation. Lowercase letters a, b, and c denote homogenous subsets where differing letters represent statistically significant differences between individual instruments (*p* < 0.05)

Seven laboratories in 4 countries, running 9 individual instruments, participated. The laboratories and their instruments are listed in Table 1. Instrument calibration was assessed by measuring the zinc concentration of the pre-diluted SRM 3168a solution (Fig. 1). Zinc concentration was 12.2 ± 0.7 µg/dL overall, and did not differ significantly (*p* = 0.8) between AAS (12.0 ± 0.5 µg/dL, *n* = 4) and ICP MS (12.4 ± 1.0 µg/dL, *n* = 4). None of the individual instruments varied more than 10% from the expected value of 12.5 µg/dL. However, differences in calibration between individual instruments were as high as 18.9% (*p* < 0.001).

**Table 1:** Participating laboratories and instruments

Institute	Location	Instrument(s)
Aga Khan University, Nutrition Research Laboratory	Karachi, Pakistan	iCE 3000 Flame AAS (Thermo Fisher Scientific, Waltham, MA)
Children’s Hospital Oakland Research Institute, UCSF Benioff Children’s Hospitals	Oakland, California, USA	5100 SVDV ICP-OES (Agilent Technologies, Santa Clara, CA)
International Centre for Diarrhoeal Disease Research	Dhaka, Bangladesh	AA-7000 Flame AAS (Shimadzu, Kyoto, Japan)
Oklahoma State University, Department of Nutritional Sciences	Stillwater, Oklahoma, USA	ELAN 9000 ICP-MS (PerkinElmer, Waltham, MA)
Laboratory of Human Nutrition; Institute of Food, Nutrition and Health; Swiss Federal Institute of Technology	Zurich, Switzerland	240FS Flame AAS (Agilent Technologies, Santa Clara, CA), iCAP RQ ICP-MS (Thermo Fisher Scientific, Waltham, MA)
Interdisciplinary Center for Plasma Mass Spectrometry; University of California, Davis	Davis, California, USA	8900 ICP-MS (Agilent Technologies, Santa Clara, CA)
Section of Pediatric Nutrition, University of Colorado School of Medicine	Aurora, Colorado, USA	AAnalyst 700 Flame AAS (PerkinElmer, Waltham, MA), 7700 ICP-MS (Agilent Technologies, Santa Clara, CA)

The pre-diluted zinc standard was measured again at the end of the analytical run and instrument drift calculated relative to the initial values. The mean drift was 0.4% ± 3.0% (*n* = 8), and did not differ significantly (*p* = 0.3) between AAS (1.8% ± 3.5%, *n* = 4) and ICP-MS-1.7% ± 1.7%, *n* = 3). One AAS instrument drifted by more than 5% from its initial values (6.9% drift, from 11.8 ± 0.53 µg/dL to 12.6 ± 0.28 µg/dL, *p* < 0.05). Data on drift were missing from one ICP-MS instrument.

**Fig. 2:**
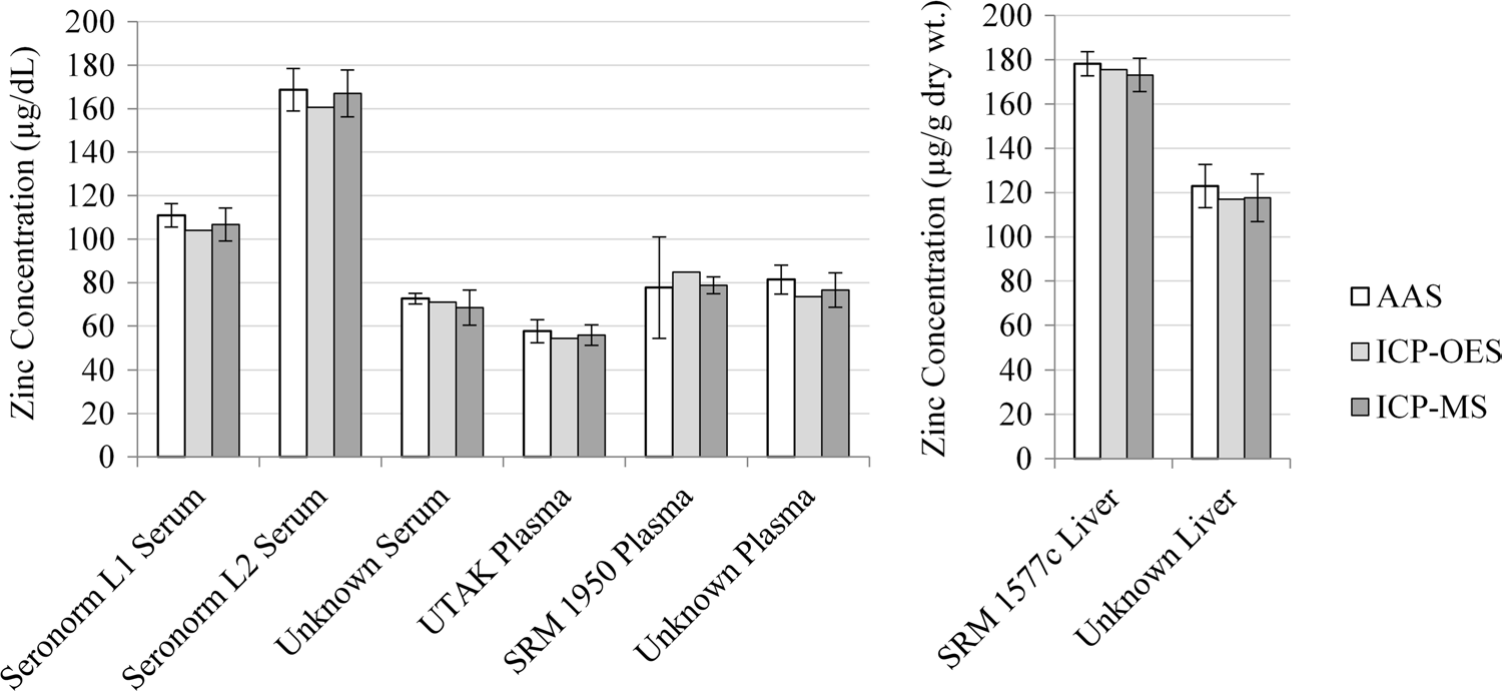
Zinc concentration of reference and unknown serum, plasma, and liver. Zinc concentration of each serum, plasma, and liver sample was determined by AAS (*n* = 4), ICP-OES (*n* = 1), and ICP-MS (*n* = 4). Data are displayed as mean ± standard deviation. No differences were detected between AAS and ICP-MS for any material, or overall

Zinc concentration of plasma, serum, and zinc content of liver samples is reported, according to each instrument type, in Fig. 2. No significant differences were observed between AAS and ICP-MS for any material measured. Precision between instrument types and samples was evaluated using the CV. The overall CV for each instrument was determined for all samples analyzed by that instrument. The instrument CV was 1.7% (0.8%, 3.7%) for AAS, 0.9% for ICP-OES, and 2.0% (1.1%, 3.4%) for ICP-MS, 1.7% (1.2%, 2.4%) overall. No differences in precision were detected between AAS and ICP-MS (*p* = 0.61, *n* = 4 each for AAS and ICP-MS).

**Fig. 3:**
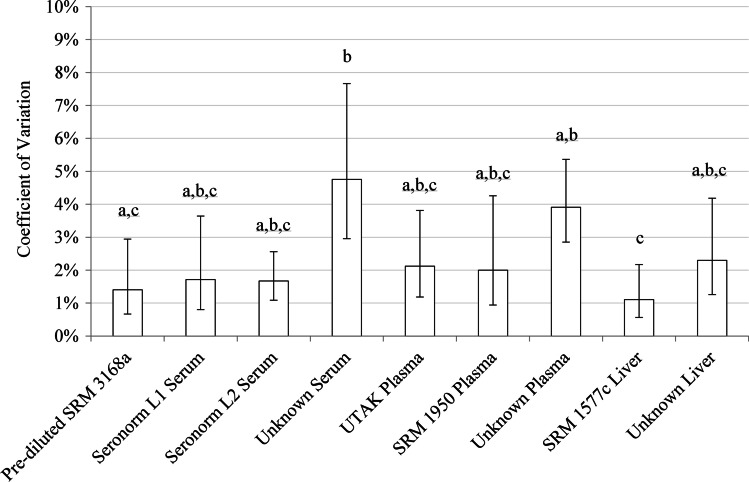
Zinc measurement precision by material analyzed. Data are expressed as geometric mean and 95% confidence interval. Lowercase letters a, b, and c denote homogenous subsets where differing letters represent statistically significant differences (*p* < 0.05)

Serum and plasma unknowns yielded higher measurement variability (lower precision) compared with other sample matrices (Fig. 3). Measures of zinc concentration of the unknown serum had a CV of 4.8% (3.0%, 7.7%), and were less precise (higher CV) than the pre-diluted SRM 3168a zinc standard solution, CV of 1.4% (0.7%, 2.9%) (*p* = 0.042), and compared with SRM 1577c liver, CV of 1.1% (0.6%, 2.2%) (*p* = 0.006). Zinc measures of unknown plasma had a CV of 3.9% (2.9%, 5.4%), and were less precise (higher) than SRM 1577c liver (*p* = 0.031).

Accuracy between instrument types and samples was evaluated by the measurement error and bias. Reference values for zinc used in calculation of accuracy were Seronorm L1 serum, 109.7 µg/dL; Seronorm L2 serum, 161.7 µg/dL; UTAK plasma, 59.4 µg/dL; SRM 1950 plasma, 71.3 µg/dL; and SRM 1577c liver, 181.1 µg/g. The reference value for SRM 1950 plasma was converted to µg/dL using the reported density of 1.02086 g/mL from the certificate of analysis.

**Fig. 4:**
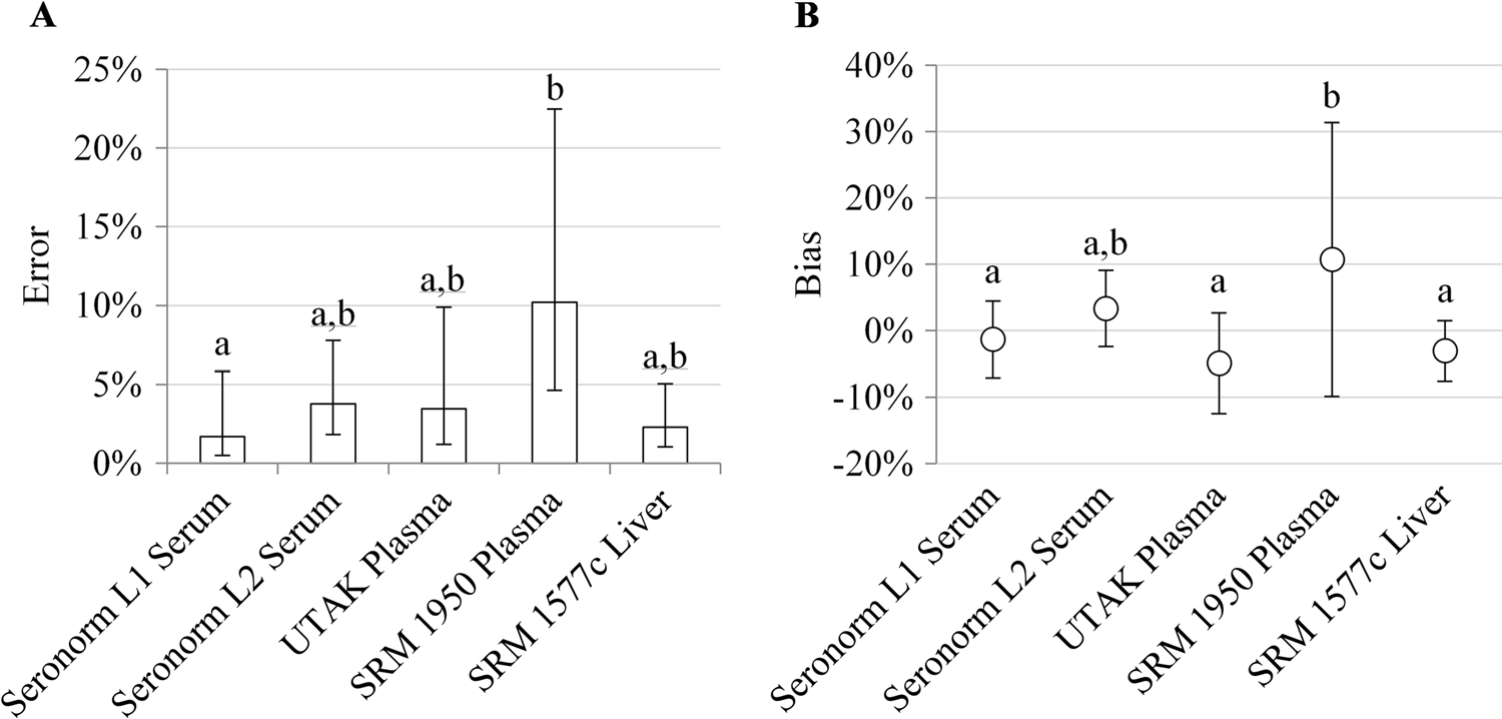
Zinc measurement accuracy by reference material analyzed. Lowercase letters a and b denote homogenous subsets where differing letters represent statistically significant differences between materials (*p* < 0.05). (A) Percent error is expressed as geometric mean and 95% CI. (B) Percent bias is expressed as mean ± SEM

Error for all instruments was 3.5% (2.2%, 5.6%) overall: 3.3% (0.8%, 14.1%) for AAS, 4.4% for ICP-OES, and 3.4% (1.8%, 6.5%) for ICP-MS. No significant difference in error was observed between AAS and ICP-MS (*p* = 0.95, *n* = 4 each). Higher error was observed with SRM 1950 plasma compared with Seronorm L1, 10.2% (4.6%, 22.5%) vs. 1.7% (0.5%, 5.8%), respectively (*p* < 0.05) (Fig. 4(A)).

The bias for all instruments was 1.0% ± 6.4% overall: 2.0% ± 9.7% for AAS, 0.3% for ICP-OES, and 0.1% ± 3.8% for ICP-MS. No significant difference in bias was observed between AAS and ICP-MS (*p* = 0.74, *n* = 4 each). Overall, measures of SRM 1950 plasma (10.8% ± 20.6%) were biased high compared with Seronorm L1 serum (− 1.3% ± 5.8%, *p* < 0.05), UTAK plasma (− 4.9% ± 7.6%, *p* < 0.01), and SRM 1577c liver (− 3.0% ± 4.6%, *p* < 0.01) (Fig. 4(B)).

## Discussion

This is the first multi-site evaluation of accuracy and precision of elemental zinc analysis in samples typical of human studies that the authors are aware of. Comparable results for the measurement of zinc were achievable with a variety of instrumentation in geographically diverse settings, using methods and samples typical of human research. However, the results for calibration, accuracy, and precision underscore potential pitfalls and areas for improvement in the implementation of zinc analytical methods in the clinical research setting.

Although differences in calibration were as high as 18% among the individual instruments, each instrument was within 10% of the expected value, an accepted cutoff for calibration verification [10]. Nonetheless, variation in the number estimated to be zinc deficient, resulting from differences in calibration, would be concerning for multi-site studies estimating population differences in zinc status. For example, consider a hypothetical population of children with a serum zinc concentration of 86 ± 13 µg/dL, and a cutoff for zinc deficiency of < 65 µg/dL. A 15% difference in calibration, where one laboratory was reporting zinc values 10% below the true value, and the other 5% above, would cause the prevalence of zinc deficiency to vary from 2 to 17%. Synchronization of instrument calibration, and verification that instruments maintain calibration over time, is essential in order to avoid such bias.

Serum or plasma zinc is the only recommended biomarker of zinc status in human populations [2]. However, in the current study, zinc measures in the donor serum and plasma yielded the lowest precision of any of the sample matrices analyzed. Although we lack data on the specific cause, one possibility is the formation of clots or precipitates in vitro. Fibrin and fibrinogen have affinity for zinc [12]. Zinc containing proteins, notably alpha-2-macroglobulin, are also present in clots [13]. Although clot formation and precipitates are causes for sample rejection for coagulation biomarkers [14], we are not aware of zinc methods specifying this rejection. We propose that our observation of higher variability in these samples warrants further study.

Care should also be taken to ensure adequate choice in reference material for verification of accuracy. For example, Seronorm Trace Elements, the most commonly mentioned reference material for serum and plasma zinc analysis based on our literature review, was available in two zinc concentration levels, i.e., 110 and 162 µg/dL. These values are substantially higher than the recommended cutoffs for zinc deficiency (i.e., from 57 to 74 µg/dL, depending on the population, fasting status, and time of collection), as well as the serum zinc concentrations of the populations from which the cutoffs were derived (ranging from 76 ± 16% to 98 ± 14% µg/dL (geometric mean ± CV)) [6, 15].

Lyophilized reference materials such as those produced by Sero and UTAK require reconstitution in the laboratory, leaving room for variability in zinc content due to variation in diluent volume or the completeness of reconstitution into solution. To avoid this inherent limitation, the authors sought a reference material that did not require reconstitution before use. NIST SRM 1950, Metabolites in Frozen Human Plasma, has a certified zinc content of 0.698 mg zinc per kilogram (71.3 µg/dL based on the certified density). This zinc concentration is also more comparable to typical cutoffs for zinc deficiency.

Surprisingly, measures of the zinc concentration in SRM 1950 had the greatest error, and were biased above the reference value. Reasons behind this finding were not determined. Although high zinc values may be due to a number of factors, they are often due to contamination. If zinc contamination were introduced in the laboratories where the vial was initially opened, it is expected that other samples handled in the same way would show a similar bias. This was not the case. The rubber stopper used in the SRM 1950 ampoule, however, may be a source of contamination. Sealing rubber typically contains zinc oxide and other zinc compounds [16], and rubber stoppers are documented contaminators of zinc and other metals in sterile pharmaceutical preparations using sealed vials similar to those used for SRM 1950 [17]. Upon their review of these data, NIST initiated the process of removing the zinc value for SRM 1950 [18].

This study has several strengths. Multiple zinc analytical laboratories were included in low- and high-income countries around the world, reflecting a global collaborative effort. A thorough literature review and input from multiple experienced zinc analytical laboratories informed the design of the laboratory activity to ensure its appropriateness. The analysis considered multiple issues, including calibration and differences by sample type. And, importantly, tight control was exerted over materials and process, so that the cause of significant differences could be effectively evaluated.

There are also several limitations to this study and the interpretation of its results. Budget limited the study to a small sample size, with only one ICP-OES instrument, limiting statistical comparisons. However, ICP-OES is also the least commonly used instrument, accounting for 15% of studies identified in our literature review, nearly half of which used the same ICP-OES instrument included in the present study. Another limitation is that calibration was only assessed at one concentration. Error in serially diluted calibrators depends on concentration, and additional concentrations would allow more precise quantification of calibration error. Future synchronization activities between laboratories should therefore include pre-diluted standards over the range of the standard curve.

Clinicians and public health scientists rely on data comparing the nutritional status of population groups to inform the design, implementation, and evaluation of nutrition interventions. Previous research has established the importance of minimizing analytical variability for biomarkers of nutritional status [19]. Studies revealing analytical variability in specific nutritional biomarkers between laboratories have led to programs for the harmonization of laboratory methods for folate [20, 21], vitamin A [22], and vitamin D [23–25]. Future studies evaluating the health consequences of zinc deficiency, as well as the impact of interventions to correct zinc deficiency, will require the comparison of zinc concentrations in serum, plasma, and food sources in diverse settings. Our results add to the justification for the further harmonization of laboratories analyzing zinc.

## Supplementary Information

Below is the link to the electronic supplementary material.

Supplementary file1 (DOCX 797 KB)

Supplementary file2 (DOCX 63.9 KB)

## Data Availability

Supporting material are available as a supplement. Data will be made available upon request to the corresponding author.
