# Supplementary material for: Comparison of Serum, Plasma, and Liver Zinc Measurements by AAS, ICP-OES, and ICP-MS in Diverse Laboratory Settings
Source: Biol Trace Elem Res. 2021 Aug 28;200(6):2606–13. doi: 10.1007/s12011-021-02883-z (PMC9132797; doi:10.1007/s12011-021-02883-z)
Supplement: Supplementary file 1 — Supplementary file1 (DOCX 797 KB) [file 12011_2021_2883_MOESM1_ESM.docx]

**Appendix A –**Comparison of serum, plasma, and liver zinc measurements by AAS, ICP-OES, and ICP-MS in diverse laboratory settings: Literature review summary

Author: Andrew G. Hall, Ph.D., aghall@berkeley.edu, aghall@ucdavis.edu

**Introduction:**

When planning this study, the authors developed a laboratory protocol to assess variability in (re-state objective of the laboratory exercise). In order to develop a perspective on what samples should be included, sample preparation method, and controls, we surveyed clinical studies over the last 5 years for their zinc analytical methodology.

**Search method:**

- Database: National Library of Medicine, PubMed
  - accessed at https://www.ncbi.nlm.nih.gov/pubmed/ on February 7, 2018
- Keyword: “Zinc” in any field
- Filters: clinical trial, published in the last 5 years, human subjects

**Results:**

470 journal articles were identified in the search. All abstracts were reviewed for zinc concentration data in human derived samples or foods, or statement that zinc concentration was measured. 142 of the 470 articles met these criteria, and were downloaded for further review of zinc methods. Four articles were excluded from further review because they were not in English, and four did not report zinc methods or data within the article. The remaining 134 papers were reviewed for specific aspects of the zinc analytical method, and are cited here *(*[*1-134*](#_ENREF_1)*)*. Methodological details were catalogued, including whether zinc analytical methods were reported, zinc method citation, sample matrices, and instrument type.

Zinc concentration was most frequently determined in serum, plasma, and food composites. Samples analyzed for zinc concentrations are listed in Table 1. Instruments used for quantification of zinc content included Atomic Absorbance Spectrometers, AAS (n = 78), Inductively Coupled Plasma Mass Spectrometers, ICP-MS (n = 22), and Inductively Coupled Plasma Optical Emissions Spectrometers, ICP‑OES (n = 16), while 7 studies used a plate reader or auto-analyzer for indirect determination of zinc concentration via a zinc sensitive chemical dye or probe (Table 2). The above total for ICP-MS does not include 14 studies where the ICP-MS instrument determined zinc isotope ratios, but another instrument (AAS or ICP-OES) was the primary instrument for quantifying zinc concentration.

For studies analyzing any of the most commonly encountered sample matrices (serum, plasma, and food composites, n = 123), data were collected on any mention in the respective publication of zinc contamination mitigation (e.g. acid washing, screening, trace element grade tubes, etc.), sample preparation method, and reference material. Only 51 of the studies described or referenced methodological measures to avoid or minimize contamination with environmental zinc, including use of zinc-free tubes, acid washing, or screening for zinc contamination.

Of the 117 studies determining serum or plasma zinc concentration, preparation by simple dilution (n = 28) and digestion before dilution (n = 21) of plasma and serum samples were reported, but plasma and serum sample preparation was frequently left unstated (n = 68). Methods for preparation of food samples for zinc analysis were specified in 11 studies, and included wet digestion using a microwave (n = 4), ashing of sample in a muffle furnace (n = 6), and wet ashing using a hot plate (n = 1).

A reference material for zinc was specified in only 34 of the 123 studies analyzing plasma, serum, or food composite zinc content. The most commonly used reference materials were Seronorm serum (n = 15), NIST SRM 1577c bovine liver (n = 5), UTAK plasma (n = 4), and NIST SRM 1598 bovine serum (n = 4).

**Table 1: Sample matrices for determination of zinc concentration**

| Sample matrix | Number of studies | Literature reference |
| --- | --- | --- |
| Serum | 69 | *(*[*1*](#_ENREF_1)*,* [*4*](#_ENREF_4)*,* [*7*](#_ENREF_7)*,* [*13*](#_ENREF_13)*,* [*15*](#_ENREF_15)*,* [*16*](#_ENREF_16)*,* [*19-24*](#_ENREF_19)*,* [*26*](#_ENREF_26)*,* [*29*](#_ENREF_29)*,* [*31*](#_ENREF_31)*,* [*32*](#_ENREF_32)*,* [*35*](#_ENREF_35)*,* [*36*](#_ENREF_36)*,* [*38*](#_ENREF_38)*,* [*41-43*](#_ENREF_41)*,* [*45-47*](#_ENREF_45)*,* [*49-53*](#_ENREF_49)*,* [*56-58*](#_ENREF_56)*,* [*63*](#_ENREF_63)*,* [*66*](#_ENREF_66)*,* [*67*](#_ENREF_67)*,* [*69*](#_ENREF_69)*,* [*71*](#_ENREF_71)*,* [*73-75*](#_ENREF_73)*,* [*78*](#_ENREF_78)*,* [*80*](#_ENREF_80)*,* [*82*](#_ENREF_82)*,* [*84-86*](#_ENREF_84)*,* [*89*](#_ENREF_89)*,* [*91*](#_ENREF_91)*,* [*92*](#_ENREF_92)*,* [*94*](#_ENREF_94)*,* [*97*](#_ENREF_97)*,* [*98*](#_ENREF_98)*,* [*100*](#_ENREF_100)*,* [*103*](#_ENREF_103)*,* [*105*](#_ENREF_105)*,* [*107*](#_ENREF_107)*,* [*108*](#_ENREF_108)*,* [*110*](#_ENREF_110)*,* [*114-116*](#_ENREF_114)*,* [*119*](#_ENREF_119)*,* [*121*](#_ENREF_121)*,* [*125*](#_ENREF_125)*,* [*127*](#_ENREF_127)*,* [*129*](#_ENREF_129)*,* [*130*](#_ENREF_130)*,* [*132*](#_ENREF_132)*)* |
| Plasma | 48 | *(*[*2*](#_ENREF_2)*,* [*3*](#_ENREF_3)*,* [*5*](#_ENREF_5)*,* [*6*](#_ENREF_6)*,* [*8-12*](#_ENREF_8)*,* [*17*](#_ENREF_17)*,* [*18*](#_ENREF_18)*,* [*25*](#_ENREF_25)*,* [*27*](#_ENREF_27)*,* [*28*](#_ENREF_28)*,* [*30*](#_ENREF_30)*,* [*33*](#_ENREF_33)*,* [*34*](#_ENREF_34)*,* [*37*](#_ENREF_37)*,* [*40*](#_ENREF_40)*,* [*48*](#_ENREF_48)*,* [*54*](#_ENREF_54)*,* [*55*](#_ENREF_55)*,* [*60-62*](#_ENREF_60)*,* [*64*](#_ENREF_64)*,* [*65*](#_ENREF_65)*,* [*68*](#_ENREF_68)*,* [*70*](#_ENREF_70)*,* [*72*](#_ENREF_72)*,* [*87*](#_ENREF_87)*,* [*88*](#_ENREF_88)*,* [*93*](#_ENREF_93)*,* [*95*](#_ENREF_95)*,* [*96*](#_ENREF_96)*,* [*99*](#_ENREF_99)*,* [*101*](#_ENREF_101)*,* [*104*](#_ENREF_104)*,* [*111*](#_ENREF_111)*,* [*112*](#_ENREF_112)*,* [*117*](#_ENREF_117)*,* [*118*](#_ENREF_118)*,* [*122*](#_ENREF_122)*,* [*123*](#_ENREF_123)*,* [*126*](#_ENREF_126)*,* [*131*](#_ENREF_131)*,* [*133*](#_ENREF_133)*,* [*134*](#_ENREF_134)*)* |
| Food composites | 12 | *(*[*14*](#_ENREF_14)*,* [*33*](#_ENREF_33)*,* [*34*](#_ENREF_34)*,* [*39*](#_ENREF_39)*,* [*65*](#_ENREF_65)*,* [*81*](#_ENREF_81)*,* [*113*](#_ENREF_113)*,* [*120*](#_ENREF_120)*,* [*122*](#_ENREF_122)*,* [*124*](#_ENREF_124)*,* [*130*](#_ENREF_130)*,* [*134*](#_ENREF_134)*)* |
| Urine | 7 | *(*[*5*](#_ENREF_5)*,* [*10*](#_ENREF_10)*,* [*11*](#_ENREF_11)*,* [*29*](#_ENREF_29)*,* [*43*](#_ENREF_43)*,* [*50*](#_ENREF_50)*,* [*56*](#_ENREF_56)*)* |
| Erythrocytes | 6 | *(*[*10*](#_ENREF_10)*,* [*56*](#_ENREF_56)*,* [*87*](#_ENREF_87)*,* [*109*](#_ENREF_109)*,* [*133*](#_ENREF_133)*,* [*134*](#_ENREF_134)*)* |
| Tissues | 4 | *(*[*59*](#_ENREF_59)*,* [*76*](#_ENREF_76)*,* [*106*](#_ENREF_106)*,* [*128*](#_ENREF_128)*)* |
| Whole blood | 3 | *(*[*83*](#_ENREF_83)*,* [*108*](#_ENREF_108)*,* [*109*](#_ENREF_109)*)* |
| Saliva | 3 | *(*[*10*](#_ENREF_10)*,* [*90*](#_ENREF_90)*,* [*102*](#_ENREF_102)*)* |
| Milk | 2 | *(*[*79*](#_ENREF_79)*,* [*130*](#_ENREF_130)*)* |
| Leukocytes | 2 | *(*[*133*](#_ENREF_133)*,* [*134*](#_ENREF_134)*)* |
| Feces | 2 | *(*[*77*](#_ENREF_77)*,* [*81*](#_ENREF_81)*)* |
| Hair | 1 | *(*[*44*](#_ENREF_44)*)* |
| Semen | 1 | *(*[*51*](#_ENREF_51)*)* |
| Cervical vaginal lavage | 1 | *(*[*101*](#_ENREF_101)*)* |

**Table 2: Instruments for quantification of zinc concentration**

|  | Number of studies | Literature reference |
| --- | --- | --- |
| Atomic Absorbance Spectrometer (AAS) | 78 | *(*[*3-7*](#_ENREF_3)*,* [*10*](#_ENREF_10)*,* [*12*](#_ENREF_12)*,* [*14-23*](#_ENREF_14)*,* [*25*](#_ENREF_25)*,* [*26*](#_ENREF_26)*,* [*30*](#_ENREF_30)*,* [*32-34*](#_ENREF_32)*,* [*36-39*](#_ENREF_36)*,* [*43*](#_ENREF_43)*,* [*45*](#_ENREF_45)*,* [*48-51*](#_ENREF_48)*,* [*54*](#_ENREF_54)*,* [*56*](#_ENREF_56)*,* [*57*](#_ENREF_57)*,* [*59-61*](#_ENREF_59)*,* [*64*](#_ENREF_64)*,* [*65*](#_ENREF_65)*,* [*68*](#_ENREF_68)*,* [*69*](#_ENREF_69)*,* [*75*](#_ENREF_75)*,* [*78-82*](#_ENREF_78)*,* [*86*](#_ENREF_86)*,* [*87*](#_ENREF_87)*,* [*89-93*](#_ENREF_89)*,* [*96-100*](#_ENREF_96)*,* [*103*](#_ENREF_103)*,* [*106*](#_ENREF_106)*,* [*107*](#_ENREF_107)*,* [*111*](#_ENREF_111)*,* [*112*](#_ENREF_112)*,* [*115-117*](#_ENREF_115)*,* [*120*](#_ENREF_120)*,* [*122-124*](#_ENREF_122)*,* [*129-133*](#_ENREF_129)*)* |
| Inductively Couple Plasma Mass Spectrometer (ICP-MS) | 22 | *(*[*2*](#_ENREF_2)*,* [*8*](#_ENREF_8)*,* [*9*](#_ENREF_9)*,* [*13*](#_ENREF_13)*,* [*27*](#_ENREF_27)*,* [*29*](#_ENREF_29)*,* [*44*](#_ENREF_44)*,* [*55*](#_ENREF_55)*,* [*58*](#_ENREF_58)*,* [*66*](#_ENREF_66)*,* [*72*](#_ENREF_72)*,* [*76*](#_ENREF_76)*,* [*77*](#_ENREF_77)*,* [*83*](#_ENREF_83)*,* [*84*](#_ENREF_84)*,* [*102*](#_ENREF_102)*,* [*104*](#_ENREF_104)*,* [*105*](#_ENREF_105)*,* [*108*](#_ENREF_108)*,* [*109*](#_ENREF_109)*,* [*126*](#_ENREF_126)*,* [*127*](#_ENREF_127)*)* |
| Inductively Coupled Plasma Optical Emission Spectrometer (ICP-OES) | 16 | *(*[*1*](#_ENREF_1)*,* [*11*](#_ENREF_11)*,* [*28*](#_ENREF_28)*,* [*41*](#_ENREF_41)*,* [*62*](#_ENREF_62)*,* [*63*](#_ENREF_63)*,* [*70*](#_ENREF_70)*,* [*88*](#_ENREF_88)*,* [*94*](#_ENREF_94)*,* [*95*](#_ENREF_95)*,* [*101*](#_ENREF_101)*,* [*113*](#_ENREF_113)*,* [*118*](#_ENREF_118)*,* [*119*](#_ENREF_119)*,* [*128*](#_ENREF_128)*,* [*134*](#_ENREF_134)*)* |
| Plate Reader or Auto-Analyzer (Indirect determination using zinc sensitive chemical dye or probe) | 7 | *(*[*47*](#_ENREF_47)*,* [*67*](#_ENREF_67)*,* [*73*](#_ENREF_73)*,* [*85*](#_ENREF_85)*,* [*121*](#_ENREF_121)*,* [*125*](#_ENREF_125)*,* [*130*](#_ENREF_130)*)* |

**References**

1. Agustina R, Bovee-Oudenhoven IM, Lukito W, Fahmida U, van de Rest O, Zimmermann MB, et al. Probiotics Lactobacillus reuteri DSM 17938 and Lactobacillus casei CRL 431 modestly increase growth, but not iron and zinc status, among Indonesian children aged 1-6 years. J Nutr 2013;143:1184-93.

2. Basu A, Betts NM, Mulugeta A, Tong C, Newman E, Lyons TJ. Green tea supplementation increases glutathione and plasma antioxidant capacity in adults with the metabolic syndrome. Nutr Res 2013;33:180-7.

3. Bertinato J, Simpson JR, Sherrard L, Taylor J, Plouffe LJ, Van Dyke D, et al. Zinc supplementation does not alter sensitive biomarkers of copper status in healthy boys. J Nutr 2013;143:284-9.

4. Bui VQ, Marcinkevage J, Ramakrishnan U, Flores-Ayala RC, Ramirez-Zea M, Villalpando S, et al. Associations among dietary zinc intakes and biomarkers of zinc status before and after a zinc supplementation program in Guatemalan schoolchildren. Food Nutr Bull 2013;34:143-50.

5. Caulfield LE, Zavaleta N, Chen P, Colombo J, Kannass K. Mineral status of non-anemic Peruvian infants taking an iron and copper syrup with or without zinc from 6 to 18 months of age: a randomized controlled trial. Nutrition 2013;29:1336-41.

6. Consolo LZ, Melnikov P, Consolo FZ, Nascimento VA, Pontes JC. Zinc supplementation in children and adolescents with acute leukemia. Eur J Clin Nutr 2013;67:1056-9.

7. Erdogan E, Canatan D, Ormeci AR, Vural H, Aylak F. The effects of chelators on zinc levels in patients with thalassemia major. J Trace Elem Med Biol 2013;27:109-11.

8. Forte G, Bocca B, Peruzzu A, Tolu F, Asara Y, Farace C, et al. Blood metals concentration in type 1 and type 2 diabetics. Biol Trace Elem Res 2013;156:79-90.

9. Foster M, Petocz P, Samman S. Inflammation markers predict zinc transporter gene expression in women with type 2 diabetes mellitus. J Nutr Biochem 2013;24:1655-61.

10. Freire SC, Fisberg M, Cozzolino SM. Dietary intervention causes redistribution of zinc in obese adolescents. Biol Trace Elem Res 2013;154:168-77.

11. Fung EB, Kwiatkowski JL, Huang JN, Gildengorin G, King JC, Vichinsky EP. Zinc supplementation improves bone density in patients with thalassemia: a double-blind, randomized, placebo-controlled trial. Am J Clin Nutr 2013;98:960-71.

12. Guo CH, Chen PC, Hsu GS, Wang CL. Zinc supplementation alters plasma aluminum and selenium status of patients undergoing dialysis: a pilot study. Nutrients 2013;5:1456-70.

13. Heidemann SM, Holubkov R, Meert KL, Dean JM, Berger J, Bell M, et al. Baseline serum concentrations of zinc, selenium, and prolactin in critically ill children. Pediatr Crit Care Med 2013;14:e202-6.

14. Kodkany BS, Bellad RM, Mahantshetti NS, Westcott JE, Krebs NF, Kemp JF, Hambidge KM. Biofortification of pearl millet with iron and zinc in a randomized controlled trial increases absorption of these minerals above physiologic requirements in young children. J Nutr 2013;143:1489-93.

15. Kolachi NF, Kazi TG, Afridi HI, Kazi NG, Mughal MA, Khan S. Effects of selenium and zinc status in biological samples of hepatitis C patient after herbal and pharmaceutical supplements. Biol Trace Elem Res 2013;152:187-94.

16. Mahdaviroshan M, Golzarand M, Taramsari MR. Effect of zinc supplementation on serum zinc and calcium levels in postmenopausal osteoporotic women in Tabriz, Islamic Republic of Iran. East Mediterr Health J 2013;19:271-5.

17. Mazani M, Argani H, Rashtchizadeh N, Ghorbanihaghjo A, Hamdi A, Estiar MA, Nezami N. Effects of zinc supplementation on antioxidant status and lipid peroxidation in hemodialysis patients. J Ren Nutr 2013;23:180-4.

18. Motswagole BS, Mongwaketse TC, Mokotedi M, Kobue-Lekalake RI, Bulawayo BT, Thomas TS, et al. The efficacy of micronutrient-fortified sorghum meal in improving the immune status of HIV-positive adults. Ann Nutr Metab 2013;62:323-30.

19. Nenni V, Nataprawira HM, Yuniati T. Role of combined zinc, vitamin A, and fish oil supplementation in childhood tuberculosis. Southeast Asian J Trop Med Public Health 2013;44:854-61.

20. Olaya GA, Lawson M, Fewtrell MS. Efficacy and safety of new complementary feeding guidelines with an emphasis on red meat consumption: a randomized trial in Bogota, Colombia. Am J Clin Nutr 2013;98:983-93.

21. Pakfetrat M, Shahroodi JR, Zolgadr AA, Larie HA, Nikoo MH, Malekmakan L. Effects of zinc supplement on plasma homocysteine level in end-stage renal disease patients: a double-blind randomized clinical trial. Biol Trace Elem Res 2013;153:11-5.

22. Pinkaew S, Winichagoon P, Hurrell RF, Wegmuller R. Extruded rice grains fortified with zinc, iron, and vitamin A increase zinc status of Thai school children when incorporated into a school lunch program. J Nutr 2013;143:362-8.

23. Prochazkova D, Jarkovsky J, Vinohradska H, Konecna P, Machacova L, Dolezel Z. Controlled diet in phenylketonuria and hyperphenylalaninemia may cause serum selenium deficiency in adult patients: the Czech experience. Biol Trace Elem Res 2013;154:178-84.

24. Radhakrishna KV, Hemalatha R, Geddam JJ, Kumar PA, Balakrishna N, Shatrugna V. Effectiveness of zinc supplementation to full term normal infants: a community based double blind, randomized, controlled, clinical trial. PLoS One 2013;8:e61486.

25. Shamim AA, Kabir A, Merrill RD, Ali H, Rashid M, Schulze K, et al. Plasma zinc, vitamin B(12) and alpha-tocopherol are positively and plasma gamma-tocopherol is negatively associated with Hb concentration in early pregnancy in north-west Bangladesh. Public Health Nutr 2013;16:1354-61.

26. Subramanyam D, Subbaiah KV, Rajendra W, Lokanatha V. Serum selenium concentration and antioxidant activity in cervical cancer patients before and after treatment. Exp Oncol 2013;35:97-100.

27. Vidovic B, Dordevic B, Milovanovic S, Skrivanj S, Pavlovic Z, Stefanovic A, Kotur-Stevuljevic J. Selenium, zinc, and copper plasma levels in patients with schizophrenia: relationship with metabolic risk factors. Biol Trace Elem Res 2013;156:22-8.

28. Wessells KR, Hess SY, Rouamba N, Ouedraogo ZP, Kellogg M, Goto R, et al. Associations between intestinal mucosal function and changes in plasma zinc concentration following zinc supplementation. J Pediatr Gastroenterol Nutr 2013;57:348-55.

29. Xu J, Zhou Q, Liu G, Tan Y, Cai L. Analysis of serum and urinal copper and zinc in Chinese northeast population with the prediabetes or diabetes with and without complications. Oxid Med Cell Longev 2013;2013:635214.

30. Zaman K, McArthur JO, Abboud MN, Ahmad ZI, Garg ML, Petocz P, Samman S. Iron supplementation decreases plasma zinc but has no effect on plasma fatty acids in non-anemic women. Nutr Res 2013;33:272-8.

31. Adriani M, Wirjatmadi B. The effect of adding zinc to vitamin A on IGF-1, bone age and linear growth in stunted children. J Trace Elem Med Biol 2014;28:431-5.

32. Argani H, Mahdavi R, Ghorbani-haghjo A, Razzaghi R, Nikniaz L, Gaemmaghami SJ. Effects of zinc supplementation on serum zinc and leptin levels, BMI, and body composition in hemodialysis patients. J Trace Elem Med Biol 2014;28:35-8.

33. Ariff S, Krebs NF, Soofi S, Westcott J, Bhatti Z, Tabassum F, Bhutta ZA. Absorbed zinc and exchangeable zinc pool size are greater in Pakistani infants receiving traditional complementary foods with zinc-fortified micronutrient powder. J Nutr 2014;144:20-6.

34. Brnic M, Wegmuller R, Zeder C, Senti G, Hurrell RF. Influence of phytase, EDTA, and polyphenols on zinc absorption in adults from porridges fortified with zinc sulfate or zinc oxide. J Nutr 2014;144:1467-73.

35. Davidson MH, Bechtel DH. Assessment of the effect of esterified propoxylated glycerol (EPG) on the status of fat-soluble vitamins and select water-soluble nutrients following dietary administration to humans for 8 weeks. Regul Toxicol Pharmacol 2014;70 Suppl 2:S143-57.

36. de Brito NJ, Rocha ED, de Araujo Silva A, Costa JB, Franca MC, das Gracas Almeida M, Brandao-Neto J. Oral zinc supplementation decreases the serum iron concentration in healthy schoolchildren: a pilot study. Nutrients 2014;6:3460-73.

37. Dias PC, Sena-Evangelista KC, Paiva MS, Ferreira DQ, Ururahy MA, Rezende AA, et al. The beneficial effects of rosuvastatin are independent of zinc supplementation in patients with atherosclerosis. J Trace Elem Med Biol 2014;28:194-9.

38. Dragutinovic VV, Tatic SB, Nikolic-Mandic SD, Tripkovic TM, Dunderovic DM, Paunovic IR. Copper as ancillary diagnostic tool in preoperative evaluation of possible papillary thyroid carcinoma in patients with benign thyroid disease. Biol Trace Elem Res 2014;160:311-5.

39. Esamai F, Liechty E, Ikemeri J, Westcott J, Kemp J, Culbertson D, et al. Zinc absorption from micronutrient powder is low but is not affected by iron in Kenyan infants. Nutrients 2014;6:5636-51.

40. Ibarra O, Gili M, Roca M, Vives M, Serrano MJ, Pareja A, et al. The Mediterranean diet and micronutrient levels in depressive patients. Nutr Hosp 2014;31:1171-5.

41. Johnson EJ, Vishwanathan R, Rasmussen HM, Lang JC. Bioavailability of AREDS1 micronutrients from softgel capsules and tablets: a pilot study. Mol Vis 2014;20:1228-42.

42. Katayama K, Saito M, Kawaguchi T, Endo R, Sawara K, Nishiguchi S, et al. Effect of zinc on liver cirrhosis with hyperammonemia: a preliminary randomized, placebo-controlled double-blind trial. Nutrition 2014;30:1409-14.

43. Kim J, Ahn J. Effect of zinc supplementation on inflammatory markers and adipokines in young obese women. Biol Trace Elem Res 2014;157:101-6.

44. Kim JE, Yoo SR, Jeong MG, Ko JY, Ro YS. Hair zinc levels and the efficacy of oral zinc supplementation in patients with atopic dermatitis. Acta Derm Venereol 2014;94:558-62.

45. Kumar A, Bagri NK, Basu S, Asthana RK. Zinc supplementation for neonatal hyperbilirubinemia: a randomized controlled trial. Indian Pediatr 2014;51:375-8.

46. Li F, Jin X, Liu B, Zhuang W, Scalabrin D. Follow-up formula consumption in 3- to 4-year-olds and respiratory infections: an RCT. Pediatrics 2014;133:e1533-40.

47. Mazani M, Fard AS, Baghi AN, Nemati A, Mogadam RA. Effect of pomegranate juice supplementation on matrix metalloproteinases 2 and 9 following exhaustive exercise in young healthy males. J Pak Med Assoc 2014;64:785-90.

48. Mendez RO, Santiago A, Yepiz-Plascencia G, Peregrino-Uriarte AB, de la Barca AM, Garcia HS. Zinc fortification decreases ZIP1 gene expression of some adolescent females with appropriate plasma zinc levels. Nutrients 2014;6:2229-39.

49. Naem NE, El-Sayed NM, Nossier SA, Abu Zeid AA. Zinc status and dietary intake of pregnant women, Alexandria, Egypt. J Egypt Public Health Assoc 2014;89:35-41.

50. Noh H, Paik HY, Kim J, Chung J. The changes of zinc transporter ZnT gene expression in response to zinc supplementation in obese women. Biol Trace Elem Res 2014;162:38-45.

51. Raigani M, Yaghmaei B, Amirjannti N, Lakpour N, Akhondi MM, Zeraati H, et al. The micronutrient supplements, zinc sulphate and folic acid, did not ameliorate sperm functional parameters in oligoasthenoteratozoospermic men. Andrologia 2014;46:956-62.

52. Sakae K, Yanagisawa H. Oral treatment of pressure ulcers with polaprezinc (zinc L-carnosine complex): 8-week open-label trial. Biol Trace Elem Res 2014;158:280-8.

53. Santucci NR, Alkhouri RH, Baker RD, Baker SS. Vitamin and zinc status pretreatment and posttreatment in patients with inflammatory bowel disease. J Pediatr Gastroenterol Nutr 2014;59:455-7.

54. Sempertegui F, Estrella B, Rodriguez O, Gomez D, Cabezas M, Salgado G, et al. Zinc as an adjunct to the treatment of severe pneumonia in Ecuadorian children: a randomized controlled trial. Am J Clin Nutr 2014;99:497-505.

55. Siega-Riz AM, Estrada Del Campo Y, Kinlaw A, Reinhart GA, Allen LH, Shahab-Ferdows S, et al. Effect of supplementation with a lipid-based nutrient supplement on the micronutrient status of children aged 6-18 months living in the rural region of Intibuca, Honduras. Paediatr Perinat Epidemiol 2014;28:245-54.

56. Suliburska J, Bogdanski P, Szulinska M, Pupek-Musialik D. The influence of antihypertensive drugs on mineral status in hypertensive patients. Eur Rev Med Pharmacol Sci 2014;18:58-65.

57. Suliburska J, Bogdanski P, Szulinska M, Pupek-Musialik D, Jablecka A. Changes in mineral status are associated with improvements in insulin sensitivity in obese patients following L-arginine supplementation. Eur J Nutr 2014;53:387-93.

58. Surono IS, Martono PD, Kameo S, Suradji EW, Koyama H. Effect of probiotic L. plantarum IS-10506 and zinc supplementation on humoral immune response and zinc status of Indonesian pre-school children. J Trace Elem Med Biol 2014;28:465-9.

59. Valenzano MC, Mercado JM, Wang X, Zurbach EP, Raines J, McDonnell E, et al. Drug delivery of zinc to Barrett's metaplasia by oral administration to Barrett's esophagus patients. Ther Deliv 2014;5:257-64.

60. Vaz-Tostes M, Viana ML, Grancieri M, Luz TC, Paula H, Pedrosa RG, Costa NM. Yacon effects in immune response and nutritional status of iron and zinc in preschool children. Nutrition 2014;30:666-72.

61. Wegmuller R, Tay F, Zeder C, Brnic M, Hurrell RF. Zinc absorption by young adults from supplemental zinc citrate is comparable with that from zinc gluconate and higher than from zinc oxide. J Nutr 2014;144:132-6.

62. Abbeddou S, Hess SY, Yakes Jimenez E, Some JW, Vosti SA, Guissou RM, et al. Comparison of methods to assess adherence to small-quantity lipid-based nutrient supplements (SQ-LNS) and dispersible tablets among young Burkinabe children participating in a community-based intervention trial. Matern Child Nutr 2015;11 Suppl 4:90-104.

63. Aydemir B, Akdemir R, Vatan MB, Cinemre FB, Cinemre H, Kiziler AR, et al. The Circulating Levels of Selenium, Zinc, Midkine, Some Inflammatory Cytokines, and Angiogenic Factors in Mitral Chordae Tendineae Rupture. Biol Trace Elem Res 2015;167:179-86.

64. Berger PK, Pollock NK, Laing EM, Chertin V, Bernard PJ, Grider A, et al. Zinc Supplementation Increases Procollagen Type 1 Amino-Terminal Propeptide in Premenarcheal Girls: A Randomized Controlled Trial. J Nutr 2015;145:2699-704.

65. Chomba E, Westcott CM, Westcott JE, Mpabalwani EM, Krebs NF, Patinkin ZW, et al. Zinc absorption from biofortified maize meets the requirements of young rural Zambian children. J Nutr 2015;145:514-9.

66. Fallah R, Sabbaghzadegan S, Karbasi SA, Binesh F. Efficacy of zinc sulfate supplement on febrile seizure recurrence prevention in children with normal serum zinc level: A randomised clinical trial. Nutrition 2015;31:1358-61.

67. Foroozanfard F, Jamilian M, Jafari Z, Khassaf A, Hosseini A, Khorammian H, Asemi Z. Effects of zinc supplementation on markers of insulin resistance and lipid profiles in women with polycystic ovary syndrome: a randomized, double-blind, placebo-controlled trial. Exp Clin Endocrinol Diabetes 2015;123:215-20.

68. Galetti V, Kujinga P, Mitchikpe CE, Zeder C, Tay F, Tossou F, et al. Efficacy of highly bioavailable zinc from fortified water: a randomized controlled trial in rural Beninese children. Am J Clin Nutr 2015;102:1238-48.

69. Habib MA, Soofi S, Sheraz A, Bhatti ZS, Okayasu H, Zaidi SZ, et al. Zinc supplementation fails to increase the immunogenicity of oral poliovirus vaccine: a randomized controlled trial. Vaccine 2015;33:819-25.

70. Hess SY, Abbeddou S, Jimenez EY, Some JW, Vosti SA, Ouedraogo ZP, et al. Small-quantity lipid-based nutrient supplements, regardless of their zinc content, increase growth and reduce the prevalence of stunting and wasting in young burkinabe children: a cluster-randomized trial. PLoS One 2015;10:e0122242.

71. Himoto T, Nomura T, Tani J, Miyoshi H, Morishita A, Yoneyama H, et al. Exacerbation of insulin resistance and hepatic steatosis deriving from zinc deficiency in patients with HCV-related chronic liver disease. Biol Trace Elem Res 2015;163:81-8.

72. Joray ML, Yu TW, Ho E, Clarke SL, Stanga Z, Gebreegziabher T, et al. Zinc supplementation reduced DNA breaks in Ethiopian women. Nutr Res 2015;35:49-55.

73. Karamali M, Heidarzadeh Z, Seifati SM, Samimi M, Tabassi Z, Hajijafari M, et al. Zinc supplementation and the effects on metabolic status in gestational diabetes: A randomized, double-blind, placebo-controlled trial. J Diabetes Complications 2015;29:1314-9.

74. Kawaguchi T, Nagao Y, Abe K, Imazeki F, Honda K, Yamasaki K, et al. Effects of branched-chain amino acids and zinc-enriched nutrients on prognosticators in HCV-infected patients: a multicenter randomized controlled trial. Mol Med Rep 2015;11:2159-66.

75. Kobayashi H, Abe M, Okada K, Tei R, Maruyama N, Kikuchi F, et al. Oral zinc supplementation reduces the erythropoietin responsiveness index in patients on hemodialysis. Nutrients 2015;7:3783-95.

76. Laine JE, Ray P, Bodnar W, Cable PH, Boggess K, Offenbacher S, Fry RC. Placental Cadmium Levels Are Associated with Increased Preeclampsia Risk. PLoS One 2015;10:e0139341.

77. Li M, Wu J, Ren T, Wang R, Li W, Piao J, et al. Effect of NaFeEDTA-fortified soy sauce on zinc absorption in children. Food Funct 2015;6:788-92.

78. Macedoni-Luksic M, Gosar D, Bjorklund G, Orazem J, Kodric J, Lesnik-Musek P, et al. Levels of metals in the blood and specific porphyrins in the urine in children with autism spectrum disorders. Biol Trace Elem Res 2015;163:2-10.

79. Mahdavi R, Taghipour S, Ostadrahimi A, Nikniaz L, Hezaveh SJ. A pilot study of synbiotic supplementation on breast milk mineral concentrations and growth of exclusively breast fed infants. J Trace Elem Med Biol 2015;30:25-9.

80. Mahmoodianfard S, Vafa M, Golgiri F, Khoshniat M, Gohari M, Solati Z, Djalali M. Effects of Zinc and Selenium Supplementation on Thyroid Function in Overweight and Obese Hypothyroid Female Patients: A Randomized Double-Blind Controlled Trial. J Am Coll Nutr 2015;34:391-9.

81. May T, Westcott C, Thakwalakwa C, Ordiz MI, Maleta K, Westcott J, et al. Resistant starch does not affect zinc homeostasis in rural Malawian children. J Trace Elem Med Biol 2015;30:43-8.

82. Mujica-Coopman MF, Borja A, Pizarro F, Olivares M. Effect of daily supplementation with iron and zinc on iron status of childbearing age women. Biol Trace Elem Res 2015;165:10-7.

83. Ng E, Lind PM, Lindgren C, Ingelsson E, Mahajan A, Morris A, Lind L. Genome-wide association study of toxic metals and trace elements reveals novel associations. Hum Mol Genet 2015;24:4739-45.

84. Paz-Tal O, Canfi A, Marko R, Katorza E, Karpas Z, Shai I, et al. Effect of changes in food groups intake on magnesium, zinc, copper, and selenium serum levels during 2 years of dietary intervention. J Am Coll Nutr 2015;34:1-14.

85. Pourhassan A, Fouladi DF, Samani SM, Asl SM. Serum Zinc and Haptoglobin in Noncirrhotic Azeri Patients with Chronic Active Hepatitis C: a Case-Control Study. Biol Trace Elem Res 2015;167:187-93.

86. Rocha ED, de Brito NJ, Dantas MM, Silva Ade A, Almeida M, Brandao-Neto J. Effect of Zinc Supplementation on GH, IGF1, IGFBP3, OCN, and ALP in Non-Zinc-Deficient Children. J Am Coll Nutr 2015;34:290-9.

87. Sena-Evangelista KC, Pedrosa LF, Paiva MS, Dias PC, Ferreira DQ, Cozzolino SM, et al. The hypolipidemic and pleiotropic effects of rosuvastatin are not enhanced by its association with zinc and selenium supplementation in coronary artery disease patients: a double blind randomized controlled study. PLoS One 2015;10:e0119830.

88. Sharif R, Thomas P, Zalewski P, Fenech M. Zinc supplementation influences genomic stability biomarkers, antioxidant activity, and zinc transporter genes in an elderly Australian population with low zinc status. Mol Nutr Food Res 2015;59:1200-12.

89. Shiota J, Tagawa H, Izumi N, Higashikawa S, Kasahara H. Effect of zinc supplementation on bone formation in hemodialysis patients with normal or low turnover bone. Ren Fail 2015;37:57-60.

90. Soares LG, Jonski G, Tinoco EM, Young A. Short-term effect of strontium- and zinc-containing toothpastes and mouthrinses on volatile sulphur compounds in morning breath: a randomized, double-blind, cross-over clinical study. Eur J Oral Sci 2015;123:72-9.

91. Solati Z, Jazayeri S, Tehrani-Doost M, Mahmoodianfard S, Gohari MR. Zinc monotherapy increases serum brain-derived neurotrophic factor (BDNF) levels and decreases depressive symptoms in overweight or obese subjects: a double-blind, randomized, placebo-controlled trial. Nutr Neurosci 2015;18:162-8.

92. Tonelli M, Wiebe N, Thompson S, Kinniburgh D, Klarenbach SW, Walsh M, et al. Trace element supplementation in hemodialysis patients: a randomized controlled trial. BMC Nephrol 2015;16:52.

93. Ahmad SM, Hossain MB, Monirujjaman M, Islam S, Huda MN, Kabir Y, et al. Maternal zinc supplementation improves hepatitis B antibody responses in infants but decreases plasma zinc level. Eur J Nutr 2016;55:1823-9.

94. Barnett JB, Dao MC, Hamer DH, Kandel R, Brandeis G, Wu D, et al. Effect of zinc supplementation on serum zinc concentration and T cell proliferation in nursing home elderly: a randomized, double-blind, placebo-controlled trial. Am J Clin Nutr 2016;103:942-51.

95. Becquey E, Ouedraogo CT, Hess SY, Rouamba N, Prince L, Ouedraogo JB, et al. Comparison of Preventive and Therapeutic Zinc Supplementation in Young Children in Burkina Faso: A Cluster-Randomized, Community-Based Trial. J Nutr 2016;146:2058-66.

96. Brnic M, Wegmuller R, Melse-Boonstra A, Stomph T, Zeder C, Tay FM, Hurrell RF. Zinc Absorption by Adults Is Similar from Intrinsically Labeled Zinc-Biofortified Rice and from Rice Fortified with Labeled Zinc Sulfate. J Nutr 2016;146:76-80.

97. Colgate ER, Haque R, Dickson DM, Carmolli MP, Mychaleckyj JC, Nayak U, et al. Delayed Dosing of Oral Rotavirus Vaccine Demonstrates Decreased Risk of Rotavirus Gastroenteritis Associated With Serum Zinc: A Randomized Controlled Trial. Clin Infect Dis 2016;63:634-41.

98. Della Lucia CM, Rodrigues KC, Rodrigues VC, Santos LL, Cardoso LM, Martino HS, et al. Diet Quality and Adequacy of Nutrients in Preschool Children: Should Rice Fortified with Micronutrients Be Included in School Meals? Nutrients 2016;8.

99. Diouf A, Badiane A, Manga NM, Idohou-Dossou N, Sow PS, Wade S. Daily consumption of ready-to-use peanut-based therapeutic food increased fat free mass, improved anemic status but has no impact on the zinc status of people living with HIV/AIDS: a randomized controlled trial. BMC Public Health 2016;16:1.

100. Elmugabil A, Hamdan HZ, Elsheikh AE, Rayis DA, Adam I, Gasim GI. Serum Calcium, Magnesium, Zinc and Copper Levels in Sudanese Women with Preeclampsia. PLoS One 2016;11:e0167495.

101. Friedland BA, Hoesley CJ, Plagianos M, Hoskin E, Zhang S, Teleshova N, et al. First-in-Human Trial of MIV-150 and Zinc Acetate Coformulated in a Carrageenan Gel: Safety, Pharmacokinetics, Acceptability, Adherence, and Pharmacodynamics. J Acquir Immune Defic Syndr 2016;73:489-96.

102. Herman M, Golasik M, Piekoszewski W, Walas S, Napierala M, Wyganowska-Swiatkowska M, et al. Essential and Toxic Metals in Oral Fluid-a Potential Role in the Diagnosis of Periodontal Diseases. Biol Trace Elem Res 2016;173:275-82.

103. Himoto T, Fujita K, Nomura T, Tani J, Miyoshi H, Morishita A, et al. Roles of Copper in Hepatocarcinogenesis via the Activation of Hypoxia-Inducible Factor-1alpha. Biol Trace Elem Res 2016;174:58-64.

104. Ho M, Heath AM, Gow M, Baur LA, Cowell CT, Samman S, Garnett SP. Zinc Intake, Zinc Bioavailability and Plasma Zinc in Obese Adolescents with Clinical Insulin Resistance Following Low Energy Diets. Ann Nutr Metab 2016;69:135-41.

105. Homan J, Schijns W, Aarts EO, van Laarhoven C, Janssen IMC, Berends FJ. An optimized multivitamin supplement lowers the number of vitamin and mineral deficiencies three years after Roux-en-Y gastric bypass: a cohort study. Surg Obes Relat Dis 2016;12:659-67.

106. Liu Z, Yang W, Long G, Wei C. Trace Elements and Chemotherapy Sensitivity. Biol Trace Elem Res 2016;173:283-90.

107. Michalska-Mosiej M, Socha K, Soroczynska J, Karpinska E, Lazarczyk B, Borawska MH. Selenium, Zinc, Copper, and Total Antioxidant Status in the Serum of Patients with Chronic Tonsillitis. Biol Trace Elem Res 2016;173:30-4.

108. Muss C, Mosgoeller W, Endler T. Prevention of "nitrosative stress" by a nutritional supplement (LaVita(R)) - a randomized placebo controlled double blind clinical trial with healthy volunteers. Neuro Endocrinol Lett 2016;37:345-52.

109. Muss C, Mosgoeller W, Endler T. Mood improving Potential of a Vitamin Trace Element Composition--A randomized, double blind, placebo controlled clinical study with healthy volunteers. Neuro Endocrinol Lett 2016;37:18-28.

110. Ruiz-Tovar J, Llavero C, Zubiaga L, Boix E. Maintenance of Multivitamin Supplements After Sleeve Gastrectomy. Obes Surg 2016;26:2324-30.

111. Suliburska J, Szulinska M, Tinkov AA, Bogdanski P. Effect of Spirulina maxima Supplementation on Calcium, Magnesium, Iron, and Zinc Status in Obese Patients with Treated Hypertension. Biol Trace Elem Res 2016;173:1-6.

112. Szymlek-Gay EA, Domellof M, Hernell O, Hurrell RF, Lind T, Lonnerdal B, et al. Mode of oral iron administration and the amount of iron habitually consumed do not affect iron absorption, systemic iron utilisation or zinc absorption in iron-sufficient infants: a randomised trial. Br J Nutr 2016;116:1046-60.

113. van der Hoeven M, Faber M, Osei J, Kruger A, Smuts CM. Effect of African leafy vegetables on the micronutrient status of mildly deficient farm-school children in South Africa: a randomized controlled study. Public Health Nutr 2016;19:935-45.

114. Wibowo N, Bardosono S, Irwinda R. Effects of Bifidobacterium animalis lactis HN019 (DR10TM), inulin, and micronutrient fortified milk on faecal DR10TM, immune markers, and maternal micronutrients among Indonesian pregnant women. Asia Pac J Clin Nutr 2016;25:S102-S10.

115. Yuan X, Qian SY, Li Z, Zhang ZZ. Effect of zinc supplementation on infants with severe pneumonia. World J Pediatr 2016;12:166-9.

116. Zhou W, Zuo X, Li J, Yu Z. Effects of nutrition intervention on the nutritional status and outcomes of pediatric patients with pneumonia. Minerva Pediatr 2016;68:5-10.

117. Ziaei S, Rahman A, Raqib R, Lonnerdal B, Ekstrom EC. A Prenatal Multiple Micronutrient Supplement Produces Higher Maternal Vitamin B-12 Concentrations and Similar Folate, Ferritin, and Zinc Concentrations as the Standard 60-mg Iron Plus 400-mug Folic Acid Supplement in Rural Bangladeshi Women. J Nutr 2016;146:2520-9.

118. Abbeddou S, Yakes Jimenez E, Some JW, Ouedraogo JB, Brown KH, Hess SY. Small-quantity lipid-based nutrient supplements containing different amounts of zinc along with diarrhea and malaria treatment increase iron and vitamin A status and reduce anemia prevalence, but do not affect zinc status in young Burkinabe children: a cluster-randomized trial. BMC Pediatr 2017;17:46.

119. Baltaci AK, Dundar TK, Aksoy F, Mogulkoc R. Changes in the Serum Levels of Trace Elements Before and After the Operation in Thyroid Cancer Patients. Biol Trace Elem Res 2017;175:57-64.

120. Dermanovic M, Miletic I, Pavlovic Z. A Comparative Analysis of the Contents Of Iron, Zinc, Copper, Manganese, and Calcium in the Collective Diet Of Preschool Children in the Northwestern Region of Bosnia. Biol Trace Elem Res 2017;175:27-32.

121. Fard FE, Mirghafourvand M, Mohammad-Alizadeh Charandabi S, Farshbaf-Khalili A, Javadzadeh Y, Asgharian H. Effects of zinc and magnesium supplements on postpartum depression and anxiety: A randomized controlled clinical trial. Women Health 2017;57:1115-28.

122. Hackl L, Speich C, Zeder C, Sanchez-Ferrer A, Adelmann H, de Pee S, et al. Cold Extrusion but Not Coating Affects Iron Bioavailability from Fortified Rice in Young Women and Is Associated with Modifications in Starch Microstructure and Mineral Retention during Cooking. J Nutr 2017;147:2319-25.

123. Hall AG, Ngu T, Nga HT, Quyen PN, Hong Anh PT, King JC. An Animal-Source Food Supplement Increases Micronutrient Intakes and Iron Status among Reproductive-Age Women in Rural Vietnam. J Nutr 2017;147:1200-7.

124. Hambidge KM, Miller LV, Mazariegos M, Westcott J, Solomons NW, Raboy V, et al. Upregulation of Zinc Absorption Matches Increases in Physiologic Requirements for Zinc in Women Consuming High- or Moderate-Phytate Diets during Late Pregnancy and Early Lactation. J Nutr 2017;147:1079-85.

125. Heidarzadeh Z, Samimi M, Seifati SM, Ashkezari MD, Ahmadi S, Mahmoodi S, et al. The Effect of Zinc Supplementation on Expressed Levels of Peroxisome Proliferator-Activated Receptor Gamma and Glucose Transporter Type 1 Genes in Newborns of Women with Gestational Diabetes Mellitus. Biol Trace Elem Res 2017;175:271-7.

126. Jobarteh ML, McArdle HJ, Holtrop G, Sise EA, Prentice AM, Moore SE. mRNA Levels of Placental Iron and Zinc Transporter Genes Are Upregulated in Gambian Women with Low Iron and Zinc Status. J Nutr 2017;147:1401-9.

127. Liu X, Piao J, Zhang Y, He Y, Li W, Yang L, Yang X. Assessment of Zinc Status in School-Age Children from Rural Areas in China Nutrition and Health Survey 2002 and 2012. Biol Trace Elem Res 2017;178:194-200.

128. Roczniak W, Brodziak-Dopierala B, Cipora E, Jakobik-Kolon A, Kluczka J, Babuska-Roczniak M. Factors that Affect the Content of Cadmium, Nickel, Copper and Zinc in Tissues of the Knee Joint. Biol Trace Elem Res 2017;178:201-9.

129. Tahmasebi K, Amani R, Nazari Z, Ahmadi K, Moazzen S, Mostafavi SA. Association of Mood Disorders with Serum Zinc Concentrations in Adolescent Female Students. Biol Trace Elem Res 2017;178:180-8.

130. Talsma EF, Moretti D, Ly SC, Dekkers R, van den Heuvel EG, Fitri A, et al. Zinc Absorption from Milk Is Affected by Dilution but Not by Thermal Processing, and Milk Enhances Absorption of Zinc from High-Phytate Rice in Young Dutch Women. J Nutr 2017;147:1086-93.

131. Uddin MG, Hossain MS, Rahman MA, Uddin A, Bhuiyan MS. Elemental Zinc Is Inversely Associated with C-Reactive Protein and Oxidative Stress in Chronic Liver Disease. Biol Trace Elem Res 2017;178:189-93.

132. Ullah Z, Ullah MI, Hussain S, Kaul H, Lone KP. Determination of Serum Trace Elements (Zn, Cu, and Fe) in Pakistani Patients with Rheumatoid Arthritis. Biol Trace Elem Res 2017;175:10-6.

133. Youssof AL, Kassim NL, Rashid SA, De Ley M, Rahman MT. Zinc Content in Cord Blood Is Associated with Maternal Age and Parity. Biol Trace Elem Res 2017;175:17-26.

134. Zyba SJ, Shenvi SV, Killilea DW, Holland TC, Kim E, Moy A, et al. A moderate increase in dietary zinc reduces DNA strand breaks in leukocytes and alters plasma proteins without changing plasma zinc concentrations. Am J Clin Nutr 2017;105:343-51.
