# Supplementary material for: Comparison of Serum, Plasma, and Liver Zinc Measurements by AAS, ICP-OES, and ICP-MS in Diverse Laboratory Settings
Source: Biol Trace Elem Res. 2021 Aug 28;200(6):2606–13. doi: 10.1007/s12011-021-02883-z (PMC9132797; doi:10.1007/s12011-021-02883-z)
Supplement: Supplementary file 2 — Supplementary file2 (DOCX 63.9 KB) [file 12011_2021_2883_MOESM2_ESM.docx]

**Table of Contents**

[**Introduction** 2](#_Toc527553912)

[**Test principles** 2](#_Toc527553913)

[**Safety** 6](#_Toc527553914)

[**Acid Washing** 6](#_Toc527553915)

[**Screening for Zinc Contamination** 8](#_Toc527553916)

[**Standards, Calibrators, and Quality Controls** 10](#_Toc527553917)

[**Preparation of Food Samples** 13](#_Toc527553918)

[**Preparation of Plasma and Serum Samples** 16](#_Toc527553919)

[**Order of Analysis and Batching, and Running the Analysis** 18](#_Toc527553920)

[**Data Evaluation and Review** 19](#_Toc527553921)

[**References** 20](#_Toc527553922)

**Introduction**

Zinc deficiency is a global health issue. Accurate and repeatable determination of the concentration of zinc in human serum or plasma, and in food samples, is essential to understanding of the extent of zinc deficiency and its causes. The measurement of zinc, although conceptually simple, is fraught with opportunity for error due to contamination from internal and external sources. Much of this potential error may be minimized, and detected when significant, with good practice in the collection, processing, and analysis of biological samples for zinc.

The IZiNCG mission is to promote and assist efforts to reduce global zinc deficiency, including the communication of analytical methods needed to fulfil these goals. Leading zinc nutrition experts from around the world were therefore contacted to formulate this set of reference methods for the analysis of zinc in human studies, with a focus on serum, plasma, and food composite zinc concentrations.

It is the purpose of this document to emphasize the methodological aspects common to zinc analysis and provide a basis for an individual laboratory to produce high quality data. Some aspects of these procedures will need to adapted and verified in the particular instrument and laboratory where they are to be applied.

**Test principles**

***Zinc detection:***

Atomic absorbance spectrometry (AAS), inductively coupled plasma optical/atomic emission spectrometry (ICP-OES or ICP-AES), and inductively coupled plasma mass spectrometry (ICP-MS) are covered here. While each has advantages and disadvantages compared to the others, all are capable of quality elemental zinc analysis. Though their sensitivities and analytical principles are different, AAS, ICP-OES, and ICP-MS have in common the introduction of liquid standards and samples into the instrument, and similar standard curve and quality control procedures.

**AAS** introduces the atomized sample into an acetylene flame or graphite furnace. The optical absorption at a wavelength for zinc is determined. This is the least expensive of the 3 methods, and the equipment is typically robust and relatively easily maintained. Interferences are in the form of spectral interferences, where signals from the combustion of other elements overlap the wavelength where zinc is determined.

**ICP-OES** introduces the atomized sample into a plasma chamber, where the sample is ionized in plasma created from an inert gas. The optical emission is then determined at a wavelength for zinc. Due to the efficient ionization of the sample, ICP-OES is generally more sensitive than AAS. Many ICP-OES instruments are equipped with multiple wavelength detectors, allowing simultaneous determination of multiple elements, and use of an internal standard to compensate for signal variation due to the instrument.

**ICP-MS** also introduces the atomized sample into a plasma chamber. However, rather than measure optical emission, a mass spectrometer uses the electric fields of ionized particles in the sample to determine the elements present. Of these methods, ICP-MS has the greatest sensitivity and is the only technique capable of determining mass. This technique is typically used where the highest sensitivity is needed (such as in the detection of toxic metals in drinking water) or where isotopic mass is needed (such as in the determination of tracer:tracee ratios in stable isotope dilution studies). ICP-MS is generally more expensive than ICP-OES, and the sensitivity of ICP-MS is greater than what is typically needed for the simple analysis of zinc concentrations in blood plasma, sera, or food samples. ICP-MS may be preferred, however, for convenience when AAS or ICP-OES instruments are not readily available.

***Standards and quality control:***

Since AAS, ICP-OES, and ICP-MS have different operational principles and sensitivities, the preparation of diluted standards for the standard curve, use of internal standards, and some aspects of quality control will differ. For example, some instruments may require analyzing a run-in of several samples to confirm that the signal has stabilized and no longer has perceptible drift that could bias the data. Matrix issues, where the viscosity of the standards needs to be matched to the viscosity of the samples (particularly with ICP-MS), or other quality control issues, may be specific to a given instrument and laboratory setup. These methods also depend on adaptation to instrument-specific operational procedures provided by the manufacturer.

The general principles of standards and quality control are, however, shared among these instruments when used for zinc analysis. A zinc standard used to make diluted calibrators is sourced from, or traceable to, the National Institute of Standards and Technology (NIST) or similar agency. Traceability to NIST provides external validity. The same diluent, made from certified trace element grade acid and ultrapure deionized water, is used to prepare calibrators and blanks, and for dilution of samples. Using the same diluent reduces the risk that a contaminated batch of diluent would go undetected and potentially bias the results. Quality control (QC) materials should be a part of every run. At a minimum these should include a pooled QC and blanks, but may also include reference materials and external zinc standards.

***Avoiding contamination:***

Zinc is ubiquitous in the environment. Sources of contamination in a clinical or laboratory environment may include dust from the atmosphere, powdered gloves, needles, tubes, and glassware.

Zinc is used in the vulcanization of rubber, so rubber stoppers typically have high amounts of zinc that can contaminate the solutions and glassware they contact. Dust can have high zinc and tubes left uncovered in a laboratory environment may quickly accumulate enough zinc from dust particles to bias the result. Glove powder typically contains zinc oxide. Handling tubes with powdered gloves can make contamination of the sample inside inevitable. Zinc can also be released from red blood cells during a blood draw or processing of serum and plasma samples.

It is recommended that the following steps be taken to avoid zinc contamination ([1-3](#_ENREF_1)):

- Blood collection tubes, needles, clot separators, glass and plastic-ware, and anything else that comes in direct contact with the sample or analytical reagents; are either certified trace-element analysis grade, pre-screened to confirm the zinc content is negligible, or acid washed to remove surface zinc contamination.
- Blood samples are collected in the morning after an overnight fast, plasma or sera separated within a half hour of collection, and samples checked for hemolysis.
- Pipettes are kept clean and calibrated. Filter pipette tips are used where appropriate to avoid contamination from the pipette mechanism. After being used once, disposable pipette tips are ejected and replaced with a new tip before pipetting the next sample.
- Ultrapure certified trace element grade acids are used. Ultrapure water is from a certified source or pre-screened for zinc content.
- Work is conducted in a clean, well-maintained laboratory environment. Contamination from dust can be minimized with the use of a clean room equipped with chemical safety fume hood, and a laminar flow biosafety cabinet, where all steps including the pipetting of acids can be safely done with minimal risk of contamination from dust. Where a clean room is not available, all steps except the pipetting of concentrated acids should be conducted in a laminar flow biosafety cabinet. An acrylic enclosure may also be substituted when working with non-biohazardous samples.
- Samples and reagents are kept covered when not in use. If an autosampler is used, it is enclosed to minimize the chance of dust entering an uncapped tube awaiting analysis.

**Safety**

***Personal protective equipment:*** For all procedures, all personnel should be equipped with lab coat, protective eyewear, and gloves.

***Human blood and blood products***: Human serum and plasma samples are potentially infectious. All handling of serum and plasma samples is to be conducted in a laminar-flow biosafety cabinet according to institutional procedures for working with human blood and blood products.

***Nitric acid***: Oxidizer, may intensify fire, causes severe skin burns and eye damage. Store away from combustible materials. Wear protective clothing, gloves, and goggles. Work with concentrated nitric acid in chemical safety fume hood.

**Acid Washing**

Acid-washing ([4](#_ENREF_4)) is an important procedural step for any containers, tubes, or pipettes that will come into contact with samples or analytical reagents, and have not been pre-screened for zinc content or otherwise verified to be free from trace metal contaminants.

***Materials***

1. Additional personal protective equipment
   1. Thick neoprene or nitrile gloves that cover the hand and forearm
   2. Laboratory goggles
2. Laboratory glassware detergent
3. Analytical grade concentrated acid (nitric acid or hydrochloric acid can be used)
4. Deionized water
5. Ultrapure water (18 MΩ.cm) for final rinse
6. Baking soda (sodium bicarbonate)
7. Plastic bins for soaking and rinsing glassware
8. Large acid-resistant covered container with mark for filling level
9. Nylon scrub brushes of appropriate sizes to clean inside glassware and/or other items to be acid-washed.

***Equipment***

1. Drying oven

***Procedure***

1. Prepare acid bath
   1. Fill large, clean acid-resistant covered container up to mark with deionized water. The filling mark should be low enough on the container that glassware can be safely retrieved from the bottom using neoprene gloves.
   2. Add concentrated acid to deionized water to make a 0.1N acid bath.
   3. Replace acid bath on a monthly basis. Neutralize acid with baking soda before discarding.
2. Prepare solution of laboratory glassware detergent and deionized water in plastic bin for soaking.
3. Soak glassware for 4 hours before scrubbing. Use nylon scrub brushes to scrub surface of glassware.
4. Rinse 5 times in deionized water.
5. Place glassware in 0.1N acid bath and soak for 12 hours (overnight).
6. Wearing nitrile or neoprene gloves that cover the forearm (over powder-free nitrile gloves), retrieve glassware and immediately rinse 5 times with ultrapure water.
7. Dry upside down on clean drying racks in drying oven at 60°C.
8. Once dry, remove the glassware, cover, and store appropriately to protect from dust.

*Notes: The effectiveness of the acid-washing procedure can be verified by rinsing the acid-washed glassware with the diluent used for blanks, and comparing the zinc content of the rinse with blank (see procedure for Screening for Zinc Contamination).*

**Screening for Zinc Contamination**

Researchers may encounter situations where it is necessary to screen for surface zinc contamination. These situations could include the necessary use of disposables that are not certified zinc-free, when verifying the effectiveness of acid-washing, or when ruling out potential sources of zinc contamination as part of troubleshooting. The procedure may vary depending on what surface is being tested for zinc contamination. The objective is to expose an ultrapure acidified diluent (the same solution used for analytical blanks) to the surface to be tested, recover the diluent, and analyze alongside a clean blank. Contamination is indicated by zinc levels above background from the blank.

***Materials***

1. Ultrapure diluent solution used for analytical blanks (i.e. made from trace metal grade concentrated acid diluted with ultrapure deionized water)
2. Zinc-free disposable transfer pipettes
3. 15 mL zinc-free conical tubes
4. Items to be tested for zinc

***Procedure***

1. Using a transfer pipette, transfer diluent to a vessel to be tested. The volume may be limited for smaller vessels, up to the volume needed for one analysis (usually 2 or 3 mL).
   1. If the item being tested is not a vessel that can hold the diluent but can fit into another clean vessel, put the item into a clean vessel and add diluent. For example, a pipette tip can be tested by placing it in a zinc-free 15 mL conical and then adding diluent.
   2. When pre-screening disposables, it is recommended to systematically select 1 of every 10 items, taking care to select from all locations within a lot.
2. Cover and swirl, shake, or vortex to give the diluent maximum exposure to the surfaces being tested.
3. Transfer the diluent from the vessel being tested to a zinc-free 15 mL conical.
4. Compare with blanks the zinc content of several tubes. Surface zinc contamination is indicated by zinc content above background.

*Notes: Reagents can also be screened for zinc content using a similar “compare with blank” procedure. For example water can be tested by adding trace element grade acid at the correct concentration for a blank, and comparing with blanks prepared using newly opened deionized water from a certified source. Acid can be tested similarly, comparing with a new bottle of trace element grade acid, each diluted to the appropriate concentration with the same deionized water.*

**Standards, Calibrators, and Quality Controls**

These components are fundamental to accuracy and validity. Extra care should be taken toward precise measurement and avoiding contamination in these steps. The zinc standard used to make the diluted calibrators is from, or traceable to, the National Institute of Standards and Technology (NIST) or similar agency. Traceability to NIST provides external validity. The same diluent is used for preparation of the calibration standards, blanks, and the dilution of samples.

The concentration of calibrators will vary depending on the instrument used. It is recommended that a standard curve comprise 4 to 6 working standard concentrations. In general, expected values of diluted unknowns should fall in the middle of the standard curve. The lowest concentration working standard should be above the detection limit for the instrument. Optimal concentrations and sample dilutions will depend on the instrument used.

Additionally, including a QC zinc standard allows verification of the diluted calibrators. This standard has a dilution that lies in the middle of the calibration curve, but is not equal to any of the calibrators. The QC standard is prepared separately from the diluted calibrators and is stored in single use aliquots ready to analyze directly. A QC standard helps in the detection of changes in calibration between runs.

QC blanks are tubes that contain diluent only. Blanks incorporated at regular intervals during a run help to detect changes in instrument background over the course of the run.

***Preparation of working standards***

This procedure is based on a commercially available zinc standard solution with concentration of 10 mg/g in 10% HNO_3_. When using other commercially available standard solutions, the procedure should be adapted according to the zinc concentration of the standard.

Materials

1. NIST SRM 3168a, 10 mg Zn / g in 10% HNO_3_, or other commercially available zinc standard solution
2. 5% HNO_3_ diluent
3. Zinc free 15 mL conical tubes
4. Zinc free polypropylene containers for storing standards
5. Zinc free filter pipette tips
6. Acid-washed volumetric flasks

Equipment

1. Calibrated pipettes
2. Precision balance
3. Vortex machine

Procedure

1. Determine the dilution values of calibrators to be used in the standard curve, and the respective amounts of diluent and concentrated working solution needed for each diluted calibrator.
2. Vortex the sealed glass vial containing the zinc standard solution. Open the vial and dispense into a zinc free 15 mL conical tube.
3. Using a precision balance and volumetric flask, make a concentrated zinc standard working solution.
   1. Weigh a capped, empty 15 mL conical and record the weight.
   2. Pipette 1 mL of SRM 3168a into the conical, and weigh. Determine the weight added by subtraction, and calculate the mass of zinc added based on the mass fraction of zinc listed in the certificate of analysis for SRM 3168a.
   3. Add 10 mL of 5% HNO_3_, cap, and vortex. Dispense into a 100 mL volumetric flask.
   4. Repeat Step “c” three times to ensure complete transfer of the zinc from the 15 mL conical to the volumetric flask. Thoroughly mix the contents of the flask.
   5. Using 5% HNO_3_, bring to a final volume of 100 mL. Transfer to a zinc free polypropylene bottle and record the zinc concentration on the bottle as (mass) Zn / (volume) 5% HNO_3_.
4. Use the working standard solution prepared in Step 2 to make calibrators volumetrically.
5. Store diluted calibrators and blank solution in zinc-free polypropylene bottles.

***Pooled Quality Controls and Reference Materials***

Internal Pooled QC materials are used to evaluate precision and accuracy both within and between runs. These are prepared from pre-screened, pooled materials of the same type being analyzed (i.e. plasma, serum, food composites, etc.) at the low-normal range for zinc content. These may additionally be spiked with known amounts of zinc to create a high-normal. Pooled QC materials are included at the beginning and end of an analytical run. Enough control material should be prepared to allow the same controls to be included in multiple analytical runs. When retiring a pool that is in low supply, the last samples from the previous pool are always included in the same run as the first samples from a new pool.

Externally produced reference QC materials with zinc values traceable to NIST may be used as a replacement for internally-verified pooled controls, or for additional verification of accuracy and precision. These may include control sera (such as UTAK or Seronorm Trace Elements in Lyophilized Serum) or NIST Standard Reference Materials (such as SRM 1577c bovine liver or SRM 1950 human plasma). When prepared from a lyophilized serum or plasma, a single batch should be produced and aliquoted at once. When going from an old lot to a new lot, or an old batch of aliquots to a new batch of aliquots, samples from the previous should be digested and analyzed in the same run with the new, as would be done with internal pooled controls.

Pooled QC materials and reference materials are measured (pipetted or weighed), digested, and diluted at the same time as the unknown samples, as outlined in the following sections.

**Preparation of Food Samples**

Dry or wet homogenous food samples can be ashed using a muffle furnace ([5](#_ENREF_5), [6](#_ENREF_6))or digested using a microwave digestion system ([7](#_ENREF_7), [8](#_ENREF_8)). If sample quantities are smaller than several grams, a microwave digestion system should be used instead of a muffle furnace to minimize the effect of contamination from the furnace environment. Pre-drying a homogenized wet sample allows reporting the results per unit dry weight. The amounts used and final dilutions will depend on the equipment used for digestion or ashing, and may be adjusted according to the needs of a particular laboratory.

***Digesting food samples using a microwave system***

The amount of sample used and the reactant may vary depending on the microwave digestion system used. These steps should be adapted according to the specific instrument setup and manufacturer-provided instructions.

Materials

1. 5% HNO_3_
2. Trace element grade concentrated HNO_3_
3. Zinc-free transfer pipettes
4. Volumetric flasks

Equipment

1. Drying oven
2. Blender
3. Precision balance

Procedure

1. Weigh ~250 mg homogenous dry powdered sample or pooled control (~500 mg undried homogenate) into acid-washed 15 mL quartz or Teflon digestion vessels in duplicate or triplicate. Record tare weight and total weight for each vessel.
2. Add 4 mL Optima concentrated HNO_3_ to each vessel.
3. Allow samples to react at room temperature for 15 minutes.
4. Digest samples using microwave digestion system according to manufacturer recommendations, using trace element grade acid.
5. Transfer digested samples into acid-washed polyethylene bottles, rinse vessels 3 times with 5% HNO_3_ and transfer rinse with the sample.
6. Using 5% HNO_3_ bring the sample to a final volume using a volumetric flask or gravimetrically on a calibrated balance.

***Ashing food samples using a muffle furnace***

Materials

1. 50 mL crucibles
2. Trace element grade concentrated HNO_3_
3. Polypropylene tubes
4. Zinc-free transfer pipettes
5. Volumetric flasks

Equipment

1. Precision balance
2. Muffle furnace
3. Drying oven (optional)
4. Hot plate
5. Fume hood

Procedure

1. Prepare an acid-washed 50 mL crucible for each sample, and record tare weight of each.
2. Weigh 5 g dry or10 g undried sample or control pool homogenate into a crucible.
3. If samples are undried, place crucibles with sample in muffle furnace or drying oven at 100°C until the weight is stable. Record the dry weight.
4. Place samples in muffle furnace. Raise the temperature slowly (~2°C to 4°C per minute) to 350°C and maintain this temperature until the samples stop releasing smoke. Slowly raise the temperature to 500°C and hold at this temperature overnight.
5. Allow crucibles to cool to room temperature.
6. In a fume hood, add 2 mL of trace element grade HNO_3_, washing any ashes from the side of the crucible as the acid is added. Place sample on a hot plate set to ~100°C until the sample is dry. Reduce the temperature if necessary to prevent boiling.
7. Place crucibles in muffle furnace overnight at 500°C for 1 hr. Repeat steps 5 – 7 until the ash appears free of carbon.
8. Completely dissolve the ash in 5.0 mL of 5% HNO_3_ and transfer to a polypropylene tube. Rinse the crucible with two additional aliquots of nitric acid, and bring to final volume using an acid washed volumetric flask or gravimetrically on a precision balance.

**Preparation of Plasma and Serum Samples**

Once collected, plasma and serum samples can be prepared for analysis in the same manner, either by diluting the sample and running directly ([4](#_ENREF_4), [9-11](#_ENREF_9)), acidifying the sample and pelleting the non-soluble portion with a centrifugation step ([12](#_ENREF_12)), or by digesting the sample with concentrated acid and heat prior to dilution and then pelleting any remaining solids ([13-15](#_ENREF_13)).

The advantage of direct dilution is the ease of maintaining volumetric precision. However the protein and fat content of the sample can also present issues of viscosity and solubility. Depending on the final dilution, direct dilution may require viscosity matching using glycerol, and may also require a detergent for the sample to stay mixed. In flame AAS, dissolved solids bound to zinc may shield the elemental zinc from the flame, leading to a lower peak optical absorption compared with the same amount of zinc in a purified standard.

One solution to these issues is to use acid to denature the proteins so they and can be pelleted and removed from the sample. This assumes that the zinc is released from the proteins that are pelleted, and thus the content of the pellet is negligible; this should be tested given the specific conditions of the protocol as implemented. Also, pelleting does not remove of the fatty portion of the sample. To prevent fats and oils in the samples from accumulating in the capillary tubes of the instrument, potentially leading to drift, a detergent may still be required.

Partial digestion of the sample using concentrated trace element grade acid and heat may acceptably reduce the issues of dissolved solids and fats even at the levels of dilution typical for AAS. However the procedure has more steps, and thus more opportunity for error, than simple dilution. Whatever method used, the processed and diluted sample, calibrators, and blanks should ultimately have equivalent concentration of acid and the same viscosity.

Materials (*these will vary depending on procedure*)

1. Zinc free 15 mL conical tubes
2. Sample diluent (in addition to deionized water, this may contain trace element grade HNO_3_, glycerol, and detergent, depending on the instrument)
3. Trace element grade concentrated HNO_3_
4. Deionized water
5. 5% HNO_3_
6. Disposable filtered pipette tips

Equipment

1. Calibrated pipetters
2. Vortex machine
3. Centrifuge

Procedure

1. Thaw serum or plasma samples and determine level of hemolysis. Generally, samples that contain hemoglobin at a concentration greater than 1 g/L are to be excluded from analysis for zinc ([3](#_ENREF_3)).
2. Check pipette calibration by pipetting water on precision balance.
3. For each sample, vortex for 3 to 5 seconds, then pipette 200 µL into a 15 mL conical.
   1. If the serum or plasma sample is partially clotted, pellet the clot by centrifugation, re-vortex and continue with pipetting, taking care to avoid the dislodged pellet or other solids present in the plasma when drawing into the pipette tip.
4. If samples are to be run directly after dilution, add the appropriate amount of sample diluent, cap tube, and vortex. For samples that are to be partially digested and remaining solids pelleted, proceed with Step 5.
5. Add 500 µL of trace element grade concentrated HNO_3_ to each tube, cap, vortex, and incubate overnight at 60°C in drying oven, with gentle shaking.
6. Dilute digested samples with 5.5 mL deionized water. This will yield an approximate concentration of 5% HNO_3_. If further dilution is needed, use 5% HNO_3_.
7. Vortex and spin at 3000 *g* for 10 min. Analyze within 1 hour.

**Order of Analysis and Batching, and Running the Analysis**

Sample should be run in the following order:

1. Calibration curve and blank,
2. QC samples and standards,
3. Unknown samples (with blank at regular intervals, e.g. every 10^th^ tube),
4. QC samples and standards (repeat).

Samples are batched in the same run according to the study design. If a subject has provided multiple samples (e.g. multiple time points), all samples from the same subject should be included in proximity in the same run to minimize within subject variability. Where multiple experimental groups are analyzed across multiple analytical runs, each run should include a similar number of tubes from each treatment group to minimize the potential for bias toward a particular experimental group. The number of samples that can be analyzed in the same run will depend on the instrument and its tendency to drift. Blanks should be run at regular intervals through the run. Quality control materials are included at the beginning and end of a run of samples. Quality control materials and samples should at a minimum be run in duplicate, and may be run in triplicate.

***Running the Analysis***

1. Vortex and spin samples within 1 hour prior to analysis.
2. Run the analysis according to manufacturer protocols for the instrument used and observe initial QC values.
   1. If pooled controls at the beginning of the run are either out of range (for commercially available controls) or vary more than 10% from the previous run, stop the analysis and troubleshoot. The analysis may be repeated once the issue has been identified and corrected, and pooled controls are within range.
3. If initial QC values are within range, continue with the analysis of remaining samples and controls.

**Data Evaluation and Review**

1. After a run, verify that quality controls are within range, and that duplicates do not exceed 10% variation.
2. If quality control materials are out of range at the beginning or end of the run, all data are rejected, the cause of error is determined, and the analysis is repeated.
3. If quality control materials exceed 10% variation between the beginning and end of the run, all data are rejected and the analysis is repeated.
4. If replicates of the same sample exceed 10%, analysis of that sample is repeated.
5. If a result falls above the range of the standard curve, the sample is diluted and analysis of that sample repeated.
6. Record the following QC data to monitor analytical performance across multiple runs:
   1. Fit of linear calibration curve
   2. QC values
   3. Variation between QC duplicates
   4. Variation between QC and beginning and end of run

**References**

1. Brown KH, Rivera JA, Bhutta Z, Gibson RS, King JC, Lonnerdal B, Ruel MT, Sandstrom B, Wasantwisut E, Hotz C. International Zinc Nutrition Consultative Group (IZiNCG) technical document #1. Assessment of the risk of zinc deficiency in populations and options for its control. Food and nutrition bulletin. 2004 Mar;25:S99-203.

2. Brown KH. IZiNCG Technical Brief No. 2: Assessing population zinc status with serum zinc concentration. Davis, California, USA: IZiNCG Secretariat; 2012.

3. IZiNCG Technical Brief No. 6: How to deal with hemolysis for plasma/serum zinc analysis. Oakland, California, USA: IZiNCG Secretariat; 2018.

4. Kemp J. Measurement of zinc concentration in plasma and serum using atomic absorption spectrophotometry. Denver, Colorado, USA: Section of Nutrition, Department of Pediatrics, University of Colorado, Denver; 2017.

5. Diet homogenization, diet samples total zinc. Denver, Colorado, USA: Section of Nutrition, Department of Pediatrics, University of Colorado, Denver.

6. Trace Metals 1. CLG-TM1.01. Washington, D.C., USA: Office of Public Health and Science, Food Safety and Inspection Service, United States Department of Agriculture; 2002.

7. Zeder C. HNL Standard Operating Procedure: Mineralization of food samples – TurboWave. Zurich, Switzerland: Laboratory of Human Nutrition at the Institute of Food, Nutrition and Health, ETH Zurich; 2014.

8. Determination of Metals by ICP-MS and ICP-OES. CLG-TM3.05. Washington, D.C., USA: Office of Public Health and Science, Food Safety and Inspection Service, United States Department of Agriculture; 2016.

9. Lei S. Measurement of zinc concentration in plasma and serum by ICP-MS. Denver, Colorado, USA: Section of Nutrition, Department of Pediatrics, University of Colorado, Denver; 2016.

10. Multi-Elements in Serum by ICP-DRC-MS. DLS 3006.8-03. Atlanta, Georgia, USA: Inorganic Radiation Analytical Toxicology, Division of Laboratory Sciences, National Center for Environmental Health, Centers for Disease Control and Prevention; 2018.

11. Stoecker BJ. Sample collection, handling, and analysis of plasma or serum by ICP-MS. Stillwater, Oklahoma, USA: Department of Nutritional Sciences, Oklahoma State University; 2018.

12. Zeder C. HNL Standard Operating Procedure: Determination of zinc concentration in serum or plasma. Zurich, Switzerland: Laboratory of Human Nutrition at the Institute of Food, Nutrition and Health, ETH Zurich; 2013.

13. Killilea DW. Laboratory protocol for the analysis of serum zinc by inductively couples plasma-optical emission spectrometry (ICP-OES). Oakland, California, USA: Children's Hospital Oakland Research Institute; 2016.

14. Killilea DW. Laboratory protocol for the analysis of plasma zinc by inductively couples plasma-optical emission spectrometry (ICP-OES). Oakland, California, USA: Children's Hospital Oakland Research Institute; 2016.

15. Jobarteh ML. Measurement of zinc concentration in plasma/serum samples using ICP-MS. Aberdeen, United Kingdom: Rowett Institute of Nutrition and Health, University of Aberdeen; 2018.
